# Soil phosphorus drives subcontinental patterns of carbon isotope discrimination across Australia

**DOI:** 10.1111/nph.71069

**Published:** 2026-03-19

**Authors:** Iftakharul Alam, Alexander W. Cheesman, Graham D. Farquhar, Thomas J. Givnish, Martin G. De Kauwe, Ernst‐Detlef Schulze, Andrea C. Westerband, Ian J. Wright, Lucas A. Cernusak

**Affiliations:** ^1^ College of Science and Engineering James Cook University Cairns Qld 4878 Australia; ^2^ Research School of Biology Australian National University Canberra ACT 2601 Australia; ^3^ Department of Botany University of Wisconsin‐Madison Madison WI 53706 USA; ^4^ School of Biological Sciences University of Bristol Bristol BS8 1TQ UK; ^5^ Max Planck Institute for Biogeochemistry Jena 07745 Germany; ^6^ Department of Biology University of Louisiana at Lafayette Lafayette LA 70503 USA; ^7^ Hawkesbury Institute for the Environment Western Sydney University Richmond NSW 2753 Australia; ^8^ School of Natural Sciences Macquarie University Sydney 2109 NSW Australia

**Keywords:** carbon isotope discrimination, leaf nitrogen, leaf phosphorus, photosynthetic capacity, stomatal conductance, water‐use efficiency

## Abstract

- Several transects have been established to study the sensitivity of carbon isotope discrimination (Δ^13^C) in woody plants to mean annual precipitation (MAP) across Australia. These have shown a surprising divergence in Δ^13^C‐MAP sensitivity among subcontinental regions.
- We analysed previously reported data alongside new measurements from a transect in northeastern Queensland to explore potential drivers of regional‐scale Δ^13^C‐MAP sensitivity.
- Multiple lines of evidence indicated this sensitivity is related to soil phosphorus. In phosphorus‐poor regions, Δ^13^C decreased less with decreasing MAP than in phosphorus‐rich regions. Along two contrasting transects in northern Australia, Δ^13^C correlated with leaf phosphorus in the phosphorus‐poor Northern Territory, but not in phosphorus‐rich northeastern Queensland, where it instead correlated with leaf nitrogen. Common garden experiments for species from phosphorus‐poor vs phosphorus‐rich regions showed contrasting relationships between Δ^13^C and species range MAP. Finally, using an Australia‐wide leaf gas exchange dataset, we showed that soil phosphorus influenced the ratio of intercellular to ambient CO_2_ concentrations (*c*_i_ : *c*_a_), which in turn controls Δ^13^C; the influence was through stomatal conductance, not photosynthetic capacity.
- Higher stomatal conductance in phosphorus‐poor regions appeared to moderate the decrease in Δ^13^C with decreasing precipitation. We suggest that high transpiration rates in these regions help to facilitate phosphorus foraging in phosphorus‐impoverished, ancient soils.

## Introduction

Along a gradient of resource availability, natural selection could be expected to favour plant ecophysiological strategies that maximise growth by improving resource‐use efficiency (Givnish, 1986; Huston, 1994). Water availability provides one such example, in which plant water‐use efficiency (WUE) typically increases in response to decreasing water availability (Ehleringer *et al*., 1992, 1998). At the leaf level, intrinsic water‐use efficiency (WUE_i_) can be defined as the ratio of photosynthesis (*A*) to stomatal conductance (*g*_s_). It provides an index of the amount of CO_2_ fixed for an amount of water that would be lost to the atmosphere at a given leaf‐to‐air vapour pressure difference. Measurements of carbon isotope discrimination (Δ^13^C) in plant dry matter can be used to infer WUE_i_, integrating over the time period during which the plant tissue was formed (Farquhar & Richards, 1984).

The term Δ^13^C describes the reduction in the ^13^C/^12^C mole fraction which takes place during photosynthesis and the ultimate conversion of atmospheric CO_2_ into plant biomass. It can be related to *c*_i_ : *c*_a_, an indicator of CO_2_ supply via stomatal conductance vs demand during photosynthesis (Farquhar *et al*., 1982, 1989):

(Eqn 1) $$\Delta^{13}C=a_{s}+\left(\bar{b}-a_{s}\right)\frac{c_{i}}{c_{a}}$$

where *a*_s_ is the ^13^C/^12^C fractionation that occurs during diffusion of CO_2_ through stomata (4.4‰), $\bar{b}$ is discrimination against ^13^CO_2_ by carboxylating enzymes (*c*. 27‰), and *c*_i_ : *c*_a_ is the ratio of intercellular to ambient CO_2_ concentrations. Eqn 1 provides a straightforward link to WUE_i_, which can also be expressed as a function of *c*_i_ : *c*_a_:

(Eqn 2) $${\mathrm{WUE}}_{i}=\frac{A}{g_{s}}=\frac{c_{a}\left(1-\frac{c_{i}}{c_{a}}\right)}{1.6}$$

where the factor 1/1.6 relates the stomatal conductance to water vapour to that for CO_2_. Thus, combining Eqns 1 and 2, it can be seen how WUE_i_ can be inferred from Δ^13^C:

(Eqn 3) $${\mathrm{WUE}}_{i}=\frac{c_{a}}{1.6}\left[1-\frac{\left(\Delta^{13}C-a_{s}\right)}{\left(\bar{b}-a_{s}\right)}\right]$$

The stable carbon isotope ratio of a plant sample is typically determined as δ^13^C, the relative deviation of its ^13^C/^12^C ratio from that of an internationally accepted standard, Vienna Pee Dee Belemnite. The Δ^13^C can be calculated from the δ^13^C of the plant sample according to the equation (Farquhar & Richards, 1984):

(Eqn 4) $$\Delta^{13}C=\frac{\delta^{13}C_{a}-\delta^{13}C_{p}}{1+\delta^{13}C_{p}}$$

where δ^13^C_a_ is the δ^13^C of atmospheric CO_2_ and δ^13^C_p_ is that of the plant sample of interest. Note that, accordingly, for plants grown under natural conditions, a δ^13^C value for atmospheric CO_2_ must be assigned to calculate Δ^13^C. As the atmosphere is generally well mixed, one approach is to assign the average δ^13^C of atmospheric CO_2_ for the year in which the sample was collected (McCarroll & Loader, 2004; Belmecheri & Lavergne, 2020; Cernusak & Ubierna, 2022). Thus, measurements of plant tissue δ^13^C can provide time‐integrated estimates *c*_i_ : *c*_a_, which relate directly to WUE_i_. In addition to ease of sample collection, Δ^13^C also offers a signal‐to‐noise ratio advantage over instantaneous gas exchange measurements, in that it integrates gas exchange variability over the period of tissue synthesis (Cernusak & Marshall, 2001; Schulze *et al*., 2014). These properties make Δ^13^C an attractive option for exploring trends in WUE_i_ across large‐scale climatic gradients.

Observations of Δ^13^C along Australian rainfall transects have shown a surprisingly large variation in terms of the change in Δ^13^C for a given change in mean annual precipitation (MAP), ranging from *c*. 1‰ m^−1^ MAP to 3‰ m^−1^ MAP (Stewart *et al*., 1995; Schulze *et al*., 1998). A similar divergence in the inferred response of water‐use efficiency also occurred if hydroclimate was instead expressed as aridity index (AI, the ratio of potential evapotranspiration to precipitation) (Miller *et al*., 2001), or as the logarithm of the ratio of MAP to mean annual pan evaporation (Givnish *et al*., 2014; Smith *et al*., 2023). One suggested explanation for regionally variable Δ^13^C‐MAP (or alternative expressions of hydroclimate) relationships in Australia is that the sensitivity of Δ^13^C to precipitation could be influenced by the seasonality of precipitation (Schulze *et al*., 1998, 2014; Cernusak *et al*., 2013). For example, the temporal distribution of rainfall in northern Australia shows a large seasonal variation compared to that in southeastern Australia, with previous studies having found contrasting responses of Δ^13^C to MAP, apparently consistent with differences in precipitation seasonality (Schulze *et al*., 1998).

Alternatively, the sensitivity of Δ^13^C to precipitation could potentially be influenced by interactions with soil nutrients. Higher soil nitrogen (N) availability is known to result in lower Δ^13^C through its influence on photosynthetic capacity (Cernusak *et al*., 2009; Garrish *et al*., 2010; Palma *et al*., 2020). While the role of phosphorus (P) has been less explored generally, global meta‐analysis has shown that both leaf P concentrations and photosynthetic capacity relate positively to soil P concentrations (Peng *et al*., 2021). Both N and P limitations in soils may lead plants to compensate for lower photosynthetic capacity by increasing intercellular CO_2_ concentrations, thereby adjusting their ratio of *A*/*g*_s_ (Givnish & Vermeij, 1976; Field *et al*., 1983; Givnish, 1986; Wright *et al*., 2001; Farquhar *et al*., 2002; Westerband *et al*., 2023). It has also been hypothesised that high transpiration rates, resulting in low WUE_i_, can transport growth‐limiting nutrients to root surfaces through mass flow of the soil solution. This argument has been made with respect to soil N (Cramer *et al*., 2008, 2009) and soil P, including organic P‐containing compounds from which phosphate could be cleaved by extracellular phosphatase enzymes released by roots into the rhizosphere (Cernusak *et al*., 2011b; Huang *et al*., 2017; Aoyagi *et al*., 2022). We suggest that this latter mechanism may be particularly relevant in Australian ecosystems, some of which can be severely impoverished with respect to soil P.

Interactions between hydroclimate, soil nutrients, and WUE_i_ may be further influenced by coordinated changes in associated plant traits (Smith *et al*., 2023; Towers *et al*., 2024). One such trait that appears to be closely linked is specific leaf area (SLA), the amount of projected leaf area for a given amount of leaf dry mass, or its inverse, leaf mass per area (LMA). In previous analyses of Δ^13^C variation among eucalypts in southwestern Australia and in a common garden in South Australia, it was concluded that responses of Δ^13^C to MAP were likely driven by associated responses of SLA to MAP, and these changes then led to the observed variations in Δ^13^C (Schulze *et al*., 2006b; Turner *et al*., 2008). In general, a response of SLA to both MAP and soil nutrient availability is predicted by theory which considers optimal resource partitioning in relation to leaf gas exchange (Givnish, 1979, 1987; Farquhar *et al*., 2002; Wright *et al*., 2003). The impact of soil nutrients, particularly soil P, in driving variations in leaf characteristics such as SLA has long been of interest in relation to the Australian flora (Beadle, 1966).

The Australian continent has remained relatively stable through geological time, with some parts not having experienced major geological disturbances even over billions of years (Djokic *et al*., 2017). Combined with continuous erosion, leaching, and fire, this has left Australian soils deprived of rock‐derived nutrients (Viscarra Rossel & Bui, 2016), resulting in some of the most P‐impoverished soils on the planet (Orians & Milewski, 2007; Kooyman *et al*., 2017). In the early stages of soil development, weathering of exposed minerals increases the amount of plant‐available soil P. As the soil matures, the amount of available P progressively declines due to the formation of insoluble minerals and organic compounds (Lambers *et al*., 2011). At the same time, there are parts of Australia, including the Great Dividing Range in eastern Australia, that are geologically younger and have been more recently shaped by tectonic activity. Along with volcanism, this has also resulted in regions of relatively higher soil P availability (Bowman, 2000). In short, the availability of soil P is expected to exert a strong selective pressure on resource uptake and utilisation strategies in Australian woody plant species, but it is yet unknown the extent to which limitations of soil P drive variation in Δ^13^C.

Our objective in this study was to gain new insight into regional‐scale drivers of sensitivity in Δ^13^C‐MAP relationships across subcontinental regions in Australia. To do this, we collated six existing datasets for the Δ^13^C of trees growing along precipitation gradients in different subcontinental regions of Australia and combined them with data for a new transect in northeastern Queensland. To standardise across datasets, we accounted for the δ^13^C of atmospheric CO_2_ in the year of sample collection and for differences in the tissue type collected (leaves vs wood). We then extracted gridded climate and soil data for sampling sites across the combined dataset and tested for detectable variation in the sensitivity of Δ^13^C to MAP among transects belonging to different regions. Following this, we tested for correlations between the regional Δ^13^C‐MAP slopes and regional edaphic and climatic characteristics, including precipitation seasonality (PS), mean annual temperature (MAT), soil N, and soil P. Upon finding that soil P was the most promising potential driver, we then examined common garden datasets and an Australia‐wide dataset of leaf gas exchange to better understand the role of soil P in modulating *c*_i_ : *c*_a_ responses to precipitation in Australian ecosystems.

## Materials and Methods

### Transects and sampling details

**Fig. 1:**
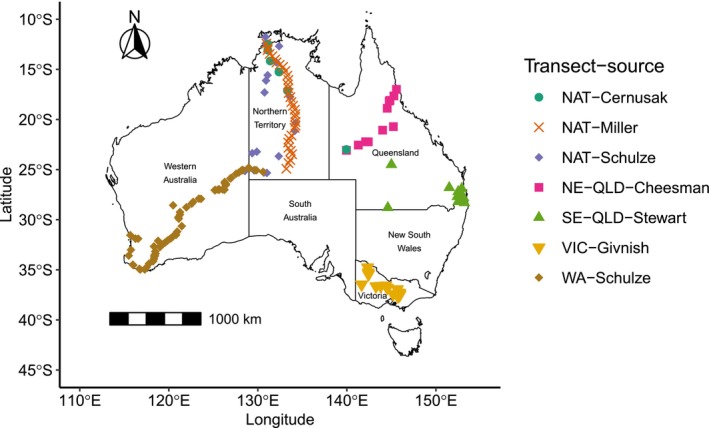
Sampling site locations and transects across Australia included in this study. Note that there are three sets of samples along a similar transect in the Northern Territory (i.e. NAT‐Cernusak, NAT‐Miller, and NAT‐Schulze), which we have retained as transect‐sources, given that sites, investigators, and years of sample collection differed.

Data including site location and plant δ^13^C from six previously published sampling transects were collated from the literature (Fig. 1; Table 1; Supporting Information Table S1) and combined with new data from a transect in northeastern Queensland. In addition to leaf or wood δ^13^C, we also collated SLA if available. All transects ranged from near the coastal regions, where MAP is generally high, to the arid interior of the Australian continent. For the new transect presented here, leaf samples were collected from trees at 11 sites in northeastern Queensland, starting near Cairns at the edge of the Australian Wet Tropics bioregion, and extending to near the edge of the Simpson Desert. Sampling from three trees of one dominant eucalypt species at each site took place during the dry season in 2015 (Cheesman & Cernusak, 2017). Data on δ^13^C, leaf nutrients, and SLA are presented here for the first time.

**Table 1:** Transect details for the combined dataset analysed in this study.

Transect	Number of sites	Sampling year	MAT (°C)	PS (%)	MAP (mm)	Soil N (t ha^−1^)	Soil P (t ha^−1^)	Sampling details	References
NAT‐Cernusak	6	2008	26.9 (24.6–27.8)	108 (83–120)	1003 (248–1658)	2.42 (1.36–3.60)	0.89 (0.63–1.40)	Leaves from two individuals of two eucalypt species per site	Cernusak *et al*. (2011a)
NAT‐Miller	33	1996	25.3 (20.5–27.7)	102 (54–123)	696 (240–1748)	2.01 (1.16–3.89)	0.78 (0.53–1.12)	Leaves from five individuals per eucalypt species, multiple species at some sites	Miller *et al*. (2001)
NAT‐Schulze	18	1993	24.9 (21.1–27.9)	88 (48–120)	674 (297–1735)	2.03 (1.11–3.82)	0.78 (0.41–1.01)	Leaves from three to five trees per species and one to 14 species per site	Schulze *et al*. (1998)
NE‐QLD‐Cheesman	11	2015	23.2 (21.4–24.8)	97 (83–110)	616 (245–1520)	2.90 (1.48–5.54)	1.90 (0.73–3.92)	Leaves from three individuals of one eucalypt species per site	Cheesman & Cernusak (2017)
SE‐QLD‐Stewart	12	1992	19.5 (17.2–22.9)	45 (37–56)	923 (323–1497)	5.33 (2.09–8.17)	1.61 (1.07–2.41)	Leaves from woody species (excluding vines), multiple species per site	Stewart *et al*. (1995)
VIC‐Givnish	20	2011	12.8 (9.6–16.9)	32 (22–41)	961 (291–1661)	5.72 (2.15–9.49)	1.28 (0.48–2.65)	Stem wood from three individuals of one eucalypt species per site	Givnish *et al*. (2014)
WA‐Schulze	74	2003	19.2 (14.6–22.9)	49 (22–85)	394 (225–1182)	2.33 (1.14–7.49)	0.70 (0.36–1.29)	Leaves from three individuals of multiple eucalypt species per site	Schulze *et al*. (2006a)

Mean transect values for all sites, along with the range in parentheses, are shown for mean annual temperature (MAT), precipitation seasonality (PS), mean annual precipitation (MAP), soil total N, and soil total P. The identities of species sampled at each site on each transect are shown in Supporting Information Table S1.

### Sample analysis

Leaf samples collected from the northeastern Queensland transect (NE‐QLD‐Cheesman) were scanned for leaf area, oven dried at 60°C, weighed for determination of SLA and then ground to a fine powder (Bench Top Ring Mill, ROCKLABS, New Zealand). Carbon isotope ratios (δ^13^C, ‰) and leaf N concentrations (mg g^−1^) were measured using an Elemental Analyser coupled via a ConFloIV to a Delta V PLUS Isotope Ratio Mass Spectrometer (ThermoScientific, Waltham, MA, USA). Leaf P concentration (mg g^−1^) was analysed by microwave‐assisted acid digestion, followed by detection via inductively coupled plasma‐atomic emission spectrometry (ICP‐AES).

Sample analysis procedures for the other transects can be found in the corresponding references shown in Table 1. For the transect in Victoria (SE‐VIC‐Givnish, Table 1), stem wood samples were collected instead of leaf samples. In order to standardise analyses across the dataset, an offset of −1.5‰ was applied to the wood δ^13^C values to make them comparable to leaf δ^13^C values (Cernusak & Ubierna, 2022). For calculating carbon isotope discrimination (Δ^13^C), the decreasing trend of δ^13^C of atmospheric CO_2_ (δ^13^C_a_) over the *c*. 30‐yr period of data collection was also accounted for, with values for the year of sample collection taken from Cernusak & Ubierna (2022).

The identities of species sampled at each site on each transect are shown in Table S1. In general, the sampling focused on eucalypts, the dominant group of tree species in Australian woodlands, including species in both the genera *Eucalyptus* and *Corymbia*. Two of the transects, NAT‐Schulze and SE‐QLD‐Stewart, also included other tree and woody species. In the case of the latter, individual species were not detailed in the original publication, and general descriptions of the vegetation communities have instead been detailed in Table S1, as originally reported.

### Gridded data layers

**Fig. 2:**
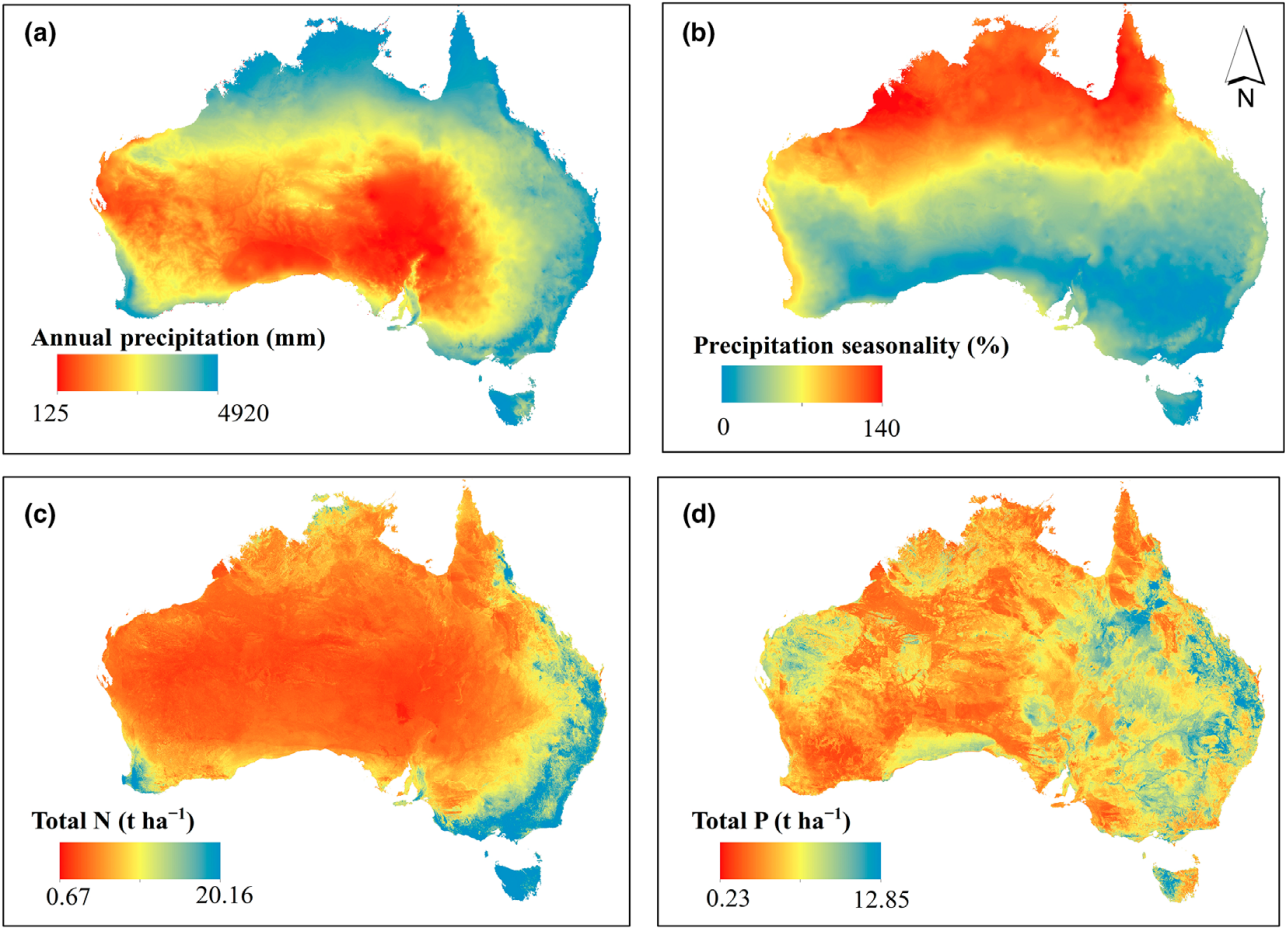
Maps showing precipitation and soil attributes across Australia: (a) mean annual precipitation (mm); (b) precipitation seasonality (coefficient of variation of monthly precipitation), where higher values indicate more seasonal precipitation and lower values indicate precipitation is spread more evenly throughout the year; (c) total nitrogen (t ha^−1^) in the 0–30 cm topsoil; and d) total phosphorus (t ha^−1^) in the 0–30 cm topsoil.

For each sampling site in the combined dataset, we extracted MAT, MAP (Fig. 2a), and PS (Fig. 2b) from the TERN Ecosystem Modelling and Scaling Infrastructure (eMAST) data products (Evans *et al*., 2015), representing a 30‐yr average (from 1976 to 2005). The PS was quantified as the SD of monthly precipitation divided by the mean (coefficient of variation), expressed as a percentage. Defined in this way, the PS can have values larger than 100%. In addition to MAP, we also considered additional metrics of hydroclimate. We extracted from the TERN eMAST data products the moisture index, defined as MAP divided by annual equilibrium evapotranspiration (Prentice *et al*., 2011), and the ratio of MAP to annual pan evaporation (Givnish *et al*., 2014). These additional metrics did not prove to be stronger predictors of Δ^13^C than MAP on its own. Thus, we retained MAP as our favoured explanatory variable, like previous analyses of Δ^13^C in Australia (Miller *et al*., 2001; Towers *et al*., 2024).

Soil total N (Fig. 2c) and soil total P (Fig. 2d) were estimated from the soil total [N] and soil total [P] datasets in the TERN Soil and Landscape Grid of Australia (Grundy *et al*., 2015), obtained at the CSIRO data access portal (Viscarra Rossel *et al*., 2014a,b; Malone & Searle, 2023). These provide Australia‐wide gridded data at *c*. 90 m × 90 m spatial resolution. We used the N and P concentrations (% soil mass) across depth increments (0–5, 5–15, and 15–30 cm) and soil bulk density (g cm^−3^) to develop depth‐weighted average topsoil (0–30 cm) total N and total P (t ha^−1^).

### Common garden experiment data

Data from two common garden studies were obtained from the literature to provide additional insight into the interaction between soil P and MAP in modulating Δ^13^C (Anderson *et al*., 1996; Cernusak, 2020). The two common garden studies provided an opportunity to contrast species that naturally occur in a higher‐P region of Australia with those that occur in a low‐P region. The first utilised 11 southeastern Australian eucalypt tree species (Anderson *et al*., 1996). We excluded two species in the dataset from Western Australia and two additional species from New South Wales, identified by the authors as phreatophytic. We used the MAP at the point of seed collection as reported in the original paper. The second study utilised nine northern Australian tree species, collected in the Northern Territory (Cernusak, 2020). In this case, the precise geographic locations of seed collection were not known; therefore, the species range MAP was used, as described in the original paper. For the species from southeastern Australia, the MAP at the species origin ranged from *c*. 200 to 1700 mm, whereas for the Northern Territory species, the species mean MAPs ranged from *c*. 500 to 1750 mm. Plants were well‐watered in both experiments when growing in the common gardens.

### Continental‐scale leaf gas exchange dataset

We used a recently compiled, Australia‐wide dataset of instantaneous measurements of leaf gas exchange to gain further insight into the relationship between *c*_i_ : *c*_a_ and soil P in Australian plants. The dataset includes measurements of 532 species from 67 sites (Westerband *et al*., 2023). Alongside *c*_i_ : *c*_a_, the dataset also contains measurements of *g*_s_ and estimates of *V*_cmax25_, the maximum Rubisco carboxylation velocity normalised to 25°C, along with estimates of MAP and soil P. We used these parameters to investigate whether the relationship between *c*_i_ : *c*_a_ and soil P was associated more strongly with stomatal conductance (*g*_s_) or with photosynthetic capacity (*V*_cmax25_). The dataset that we analysed contains *c*. 8% more species by site values for these parameters than originally presented by Westerband *et al*. (2023), due to the inclusion of some additional, previously unpublished data. The updated dataset is publicly available as described in the Data availability statement of this paper.

### Statistical methods

To aid physiological interpretation, we calculated Δ^13^C‐derived *c*_i_ : *c*_a_ according to Eqn 1 and plotted this in graphical representations. However, we fitted all statistical models using Δ^13^C, as it is not bounded between 0 and 1 and exhibits homogeneous residual variance. The Δ^13^C‐derived *c*_i_ : *c*_a_ represents a linear transformation of Δ^13^C, such that a positive trend in Δ^13^C equates to a positive trend in Δ^13^C‐derived *c*_i_ : *c*_a_.

We used analysis of covariance (ancova) to test whether Δ^13^C‐MAP slopes differed among subcontinental regions within Australia, represented by the different transects. Because the number of sites sampled within a transect varied broadly (Table 1), sites within each 100 mm MAP interval were binned before further analysis, to better balance the representation of the transects in the combined dataset. Within each 100 mm MAP interval, the gridded site data, as well as the Δ^13^C and SLA, were averaged.

For the ancova, we used a linear mixed‐effects model to test whether slopes of the relationship between Δ^13^C and the natural logarithm of MAP, log_e_(MAP), differed among transects. We applied the natural logarithm transformation to MAP in all analyses in which we used it as a predictor. The fixed effects in the linear model included log_e_(MAP), transect id, and their interaction. The data source was taken as a random effect. The NAT transect in the Northern Territory was considered a single transect, which had three data sources (NAT‐Cernusak, NAT‐Miller, and NAT‐Schulze) with random intercepts in the model. No other transects had multiple data sources. We considered a significant interaction term between log_e_(MAP) and transect id as evidence of different Δ^13^C‐log_e_(MAP) slopes among transects. The linear mixed‐effects model was fit using the *glmmTMB* package in R (Brooks *et al*., 2017). To account for unequal variance among transects, we modelled the dispersion parameter as a function of transect (dispformula = ~ transect in *glmmTMB*). Interaction and main effects were summarised using Type III Anova with Wald chi‐square tests in the *car* package (Fox & Weisberg, 2019). This analysis was also repeated with SLA to examine its variation in relation to MAP. We further fit a linear ANCOVA model with transect‐source in place of transect, such that separate slope estimates were obtained for the three NAT transect data sources. We then used the *emmeans* package (Lenth, 2024) to extract the Δ^13^C‐log_e_(MAP) slopes. Following this, transect‐wide averages of PS, MAT, soil N, and soil P were calculated as the average of the sites across each transect source. We retained the different data sources within the NAT transect in this step, given the potential for different soil N and P associated with the different sets of sampling sites. The Δ^13^C‐log_e_(MAP) slopes among transect‐sources were then tested for correlations with transect‐source‐wide averages of the predictors.

In addition to the two‐stage analysis described earlier, we also conducted analyses using stepwise regression of all site‐level data based on the Akaike Information Criterion (AIC). Forward and backward selection were applied iteratively using the *step()* function in the base R *stats* package. Starting with a null model (intercept‐only) and the full possible model, candidate predictors were added or removed to minimise the AIC, and the final model was selected as the one with the lowest AIC. Candidate predictors for the response variable Δ^13^C included log_e_(MAP), MAT, PS, log_e_(soil N), and log_e_(soil P). A correlation matrix showing relationships among these predictors is presented in Fig. S1.

Following these analyses, two transects in northern Australia (i.e. NAT‐Cernusak and NE‐QLD‐Cheesman) were examined in greater detail as both leaf N and leaf P concentrations were available for the leaf dry matter of the trees sampled on these transects (but not for the other transects). This comparison was particularly informative because the two transects have similar MAT and PS, but different soil P (Table 1; Fig. 2). Partial regression was used to tease apart the dominant nutritional driver, leaf N or P, of Δ^13^C on the two transects. Partial regression plots provided a means to visualise the unique contributions of independent variables in the multiple regression analyses. In this case, leaf Δ^13^C was the response variable and leaf N and leaf P were independent variables. We reported beta values (β) for each predictor, representing regression weights for standardised variables, from the partial regression analyses. These indicate the change in the response variable in SD associated with a change of one SD in a predictor, while holding other predictors constant. Partial regression analyses were conducted using the R package *visreg* (Breheny & Burchett, 2017), and β values were obtained from the R package *report* (Makowski *et al*., 2023).

We used a similar approach for examining the Australia‐wide leaf gas exchange dataset of Westerband *et al*. (2023). We tested whether patterns of *c*_i_ : *c*_a_, as determined by instantaneous gas exchange measurements, in response to site‐level variation in soil P were driven either by maximum carboxylation capacity (*V*_cmax25_) or by stomatal conductance (*g*_s_). Site‐averaged data were used for the analyses with this dataset. For the two common garden datasets, we tested for significant correlations between leaf Δ^13^C and the MAP at the sites of origin. Analyses were conducted in R 4.4.2 (R Core Team, 2024). Graphs were produced with *ggplot2* (Wickham, 2016). Maps for Fig. 2 were created in ArcMap v.10.8 (ESRI, Redlands, CA, USA).

## Results

### Transect characteristics

The sampling transects combined here ranged in length from *c*. 400 to 2500 km, with estimated MAP across transects typically ranging from *c*. 300 to 1700 mm (Table 1; Fig. 2a). The MAT varies latitudinally, with the highest values on the transects in northern Australia, and the lowest values on the Victorian transect (VIC‐Givnish) (Table 1). As seen in Fig. 2(b), precipitation is highly seasonal across northern Australia, moderately seasonal in southwestern Australia, and nearly aseasonal in southeastern Australia, with transects distributed across nearly this full range of variation (Table 1).

Soil N is generally higher in areas of higher rainfall across the continent (Fig. 2). Mean values were highest on the Victoria transect (VIC‐Givnish) and the southeastern Queensland transect (SE‐QLD‐Stewart) (Table 1; Fig. 2c). Mean values were generally lower in the Northern Territory (NAT‐Cernusak, NAT‐Miller, and NAT‐Schulze) and southwestern Australia (WA‐Schulze) (Table 1; Fig. 2c).

Generally, eastern Australia has higher soil P compared to the western part of the continent (Fig. 2d). Higher estimates for soil P were found in northeastern Queensland (NE‐QLD‐Cheesman), southeastern Queensland (SE‐QLD‐Steward), and Victoria (VIC‐Givnish) (Table 1; Fig. 2d). Transects in southwestern Australia and the Northern Territory had markedly lower ranges of soil P. Among the transects in northern Australia, the northeastern Queensland transect (NE‐QLD‐Cheesman) follows a path of consistently higher soil P compared to the Northern Territory transect (Table 1; Fig. 2d).

### Relationships between Δ^13^C and MAP

**Fig. 3:**
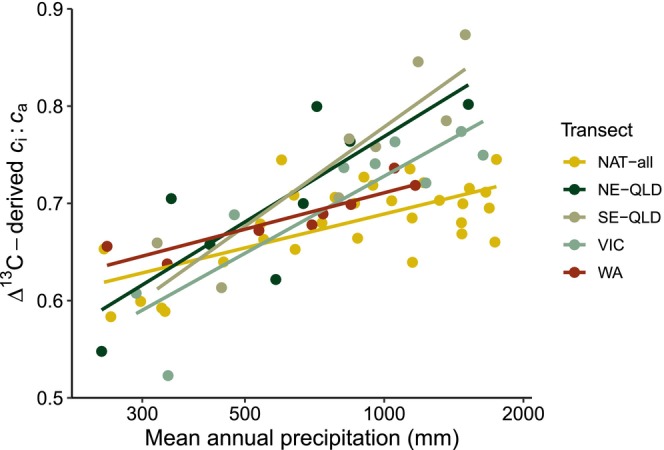
Relationships between site‐averaged leaf Δ^13^C–derived *c*_i_ : *c*_a_ and mean annual precipitation (MAP) for the studied transects. The solid lines show modelled fits for each transect. The three sources of data for the NAT transect were included in the model as a random effect. The fixed model effects are shown in the figure. Note that the scale on the *x*‐axis is logarithmic (natural logarithm).

For the transect presented for the first time here (NE‐QLD‐Cheesman), MAP varied from 235 to 1388 mm across 11 sampling locations stretching across a 900 km gradient. The Δ^13^C increased with increasing MAP, with values for individual trees ranging from 16.3‰ to 23.4‰. In the comparison between Δ^13^C and MAP across transects, it is apparent that all three eastern Australian transects (i.e. NE‐QLD‐Cheesman, SE‐QLD‐Stewart, VIC‐Givnish) have steeper Δ^13^C‐MAP slopes compared to the northern and western Australian transects (Figs 3, S2; Table 2). The linear mixed‐effects model showed significant effects on Δ^13^C of log_e_(MAP) (χ^2^(1) = 77.4, *P* < 0.001), transect id ((χ^2^(4) = 15.2, *P* = 0.004)) and of their interaction (χ^2^(4) = 18.1, *P* = 0.001). The significant interaction indicated significant variation among transects in their Δ^13^C‐log_e_(MAP) slopes. The estimates for the Δ^13^C‐log_e_(MAP) slopes for the individual transects are provided in Table 2, along with their 95% confidence intervals. We included in Table 2 the slopes obtained for the three different sources of data for the NAT transect, so that variation among these can also be seen. The slopes shown in Table 2 are the same as those that would be obtained by bivariate regression of each transect individually, but the confidence intervals differ due to the model structure, which included a dispersion sub‐model to account for heteroskedastic residuals. Overall, the variation among transects in Δ^13^C or Δ^13^C–derived *c*_i_ : *c*_a_ was most apparent at high values of MAP (Figs S2, 3). At low values of MAP, these quantities generally converged among transects.

**Table 2:** Slope estimates between leaf Δ^13^C and the natural logarithm of mean annual precipitation for each transect and transect‐source for the NAT transect.

Identifier	Transect name	Slope	SE	Lower CL	Upper CL
1	NAT‐Cernusak	0.47	0.42	−0.39	1.32
2	NAT‐Miller	1.66	0.20	1.25	2.08
3	NAT‐Schulze	0.86	0.32	0.22	1.50
4	NE‐QLD‐Cheesman	2.87	0.74	1.37	4.36
5	SE‐QLD‐Stewart	3.33	0.59	2.15	4.51
6	VIC‐Givnish	2.59	0.49	1.60	3.58
7	WA‐Schulze	1.22	0.21	0.80	1.64

Also shown are the SE of each slope, and the lower and upper confidence limits (CL) defining the 95% confidence intervals. The slope estimates, SE, and confidence intervals were derived from a linear model that included an interaction effect between transect‐source and log_e_(MAP) and a dispersion sub‐model to account for unequal variance among transects.

### Regional variation in slopes between Δ^13^C and MAP

**Fig. 4:**
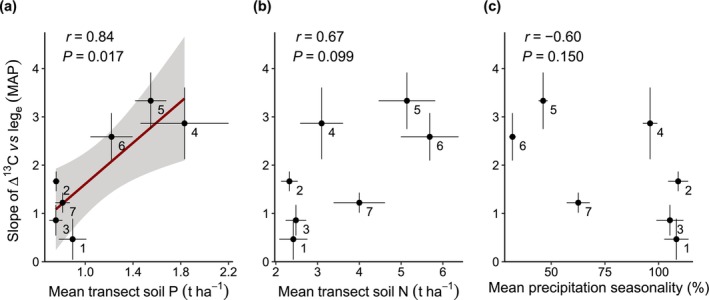
Variation in slope values for the relationship between Δ^13^C and the natural logarithm of mean annual precipitation, log_e_(MAP), for individual transects plotted against transect‐wide means of (a) soil P, (b) soil N, and (c) precipitation seasonality. Each point represents a single transect. Error bars represent SE. The solid red line in panel a shows a linear regression fit and the grey shaded area is the 95% confidence interval around the regression. The number next to each point corresponds to the transect‐source identifier in Table 2. The Pearson correlation coefficient and associated *P*‐value are shown in the top left of each panel.

Mean transect values of soil P, soil N, PS, and MAT were tested for correlations with the Δ^13^C‐log_e_(MAP) slopes shown in Table 2. Mean soil P had a significant, positive correlation with Δ^13^C‐log_e_(MAP) slopes, as shown in Fig. 4(a). Mean soil N also showed a positive correlation with Δ^13^C‐log_e_(MAP) slopes, but the bivariate correlation was not as strong as that with soil P, and it was not significant (Fig. 4b). Mean PS had a negative correlation with Δ^13^C‐log_e_(MAP) slopes, but it also was not significant (Fig. 4c). MAT had the weakest correlation with Δ^13^C‐log_e_(MAP) slopes (*r* = −0.51, *P* = 0.24). An AIC‐based stepwise model selection starting with soil P, soil N, PS, and MAT also indicated that soil P and soil N best explained regional variation in Δ^13^C‐log_e_(MAP) slopes. In the favoured model, soil P and soil N together explained 83% of the variation in slopes across transects (adjusted *R* ^2^ = 0.74, *F*_2,4_ = 9.62, *P* = 0.03). Soil P was a significant predictor in the multiple regression (std. β = 0.68, *P* = 0.041), whereas soil N was not (std. β = 0.38, *P* = 0.173); however, soil N was retained in the best AIC‐based model, indicating the importance of both nutrients in influencing regional relationships between Δ^13^C and MAP.

To complement the above two‐stage analysis, we also conducted an AIC‐based stepwise regression using all site‐level data. We used Δ^13^C as the response variable, and log_e_(MAP), log_e_(soil P), log_e_(soil N), PS, and MAT, in addition to all possible interactions, as candidate predictors. Forward and backward selection resulted in a model that retained log_e_(MAP), log_e_(soil P), and their interaction. The model explained 45% of variation in site‐level Δ^13^C (adjusted *R* ^2^ = 0.44, *F*_3,170_ = 45.7, *P* < 0.001). Both log_e_(MAP) and log_e_(soil P) were significant as main effects (*F*_1,170_ = 90.4, *P* < 0.001, η^2^_p_ = 0.35 and *F*_1,170_ = 5.2, *P* = 0.02, η^2^_p_ = 0.03, respectively), and their interaction was also significant (*F*_1,170_ = 6.8, *P* = 0.01, η^2^_p_ = 0.04). This reinforced the results of our two‐stage analysis in identifying soil P as the most important driver in modulating the relationship between Δ^13^C and MAP. The significant interaction between log_e_(MAP) and log_e_(soil P) is further visualised in a three‐dimensional perspective plot in Fig. S3.

### Covariation between SLA or LMA and Δ^13^C

We examined the relationship between SLA and MAP to assess the extent to which SLA might mediate regional variation in Δ^13^C‐log_e_(MAP) slopes. First, we fitted a linear mixed‐effects model with SLA as the response variable and log_e_(MAP), transect id, and their interaction as fixed effects. The source of data for the transects was again taken as a random effect. We used binned data, similar to the analysis shown in Fig. 3. As seen in Fig. S4, log_e_(MAP) was a significant and positive predictor of SLA (χ^2^(1) = 38.3, *P* < 0.001). However, unlike the case for Δ^13^C or Δ^13^C–derived *c*_i_ : *c*_a_, the interaction between log_e_(MAP) and transect id was not significant, indicating that SLA‐log_e_(MAP) slopes did not differ among transects (χ^2^(3) = 5.1, *P* = 0.16); transect id also was not significant as a main effect (χ^2^(3) = 4.7, *P* = 0.19). As noted, we lacked SLA data for the SE‐QLD‐Stewart transect. However, this analysis based on four transects for which we were able to compile data did not support the idea that SLA in and of itself was likely the primary driver of variation in Δ^13^C‐log_e_(MAP) slopes among transects, because SLA‐log_e_(MAP) slopes themselves did not differ.

On the other hand, across all site‐level data, we observed a clear association between Δ^13^C and SLA. The relationship was slightly improved by using LMA instead of SLA. This relationship between Δ^13^C and LMA is shown in Fig. S5, in which LMA explained 34% of the variation in site‐averaged Δ^13^C. To gain further insight, we fitted a multiple regression model using all site‐level data with Δ^13^C as the response variable and LMA, log_e_(soil P), and their interaction as predictors. This showed both LMA and log_e_(soil P) to be significant predictors of Δ^13^C (*F*_1,154_ = 61.5, *P* < 0.001, η^2^_p_ = 0.29 and *F*_1,154_ = 5.7, *P* = 0.02, η^2^_p_ = 0.04, respectively), and their interaction was significant (*F*_1,154_ = 5.4, *P* = 0.02, η^2^_p_ = 0.03). This analysis is visualised in Fig. S6. It demonstrates that soil P and its interaction with LMA explain variation in Δ^13^C additional to that explained only by LMA.

### Leaf N and P on two contrasting transects in northern Australia

**Fig. 5:**
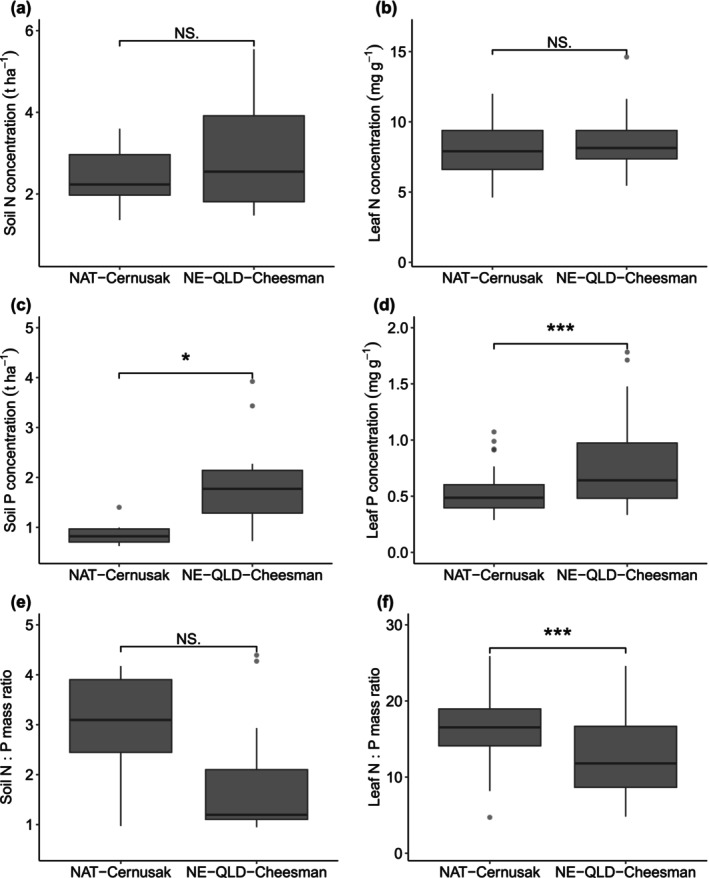
Boxplots of soil and leaf nitrogen (N) and phosphorus (P) for transects in the Northern Territory (NAT‐Cernusak) and northeastern Queensland (NE‐QLD‐Cheesman). Panels (a, c, e) show estimates of soil [N], soil [P], and soil N : P, respectively, for the sampling sites from the two transects. Panels (b, d, f) show leaf [N], leaf [P], and leaf N : P for trees from the two transects, respectively. Significance terms in the panels are based on *t*‐tests, with notation as follows: ns, not significant, *, *P* ≤ 0.05; ***, *P* ≤ 0.001. Boxes show the interquartile ranges, thick horizontal lines the medians, and whiskers the ranges of data within 1.5 times the interquartile ranges. Data points beyond the whiskers represent outlying points. For panels (a, c, e), *n* = 6 for NAT‐Cernusak and *n* = 11 for NE‐QLD‐Cheesman. For panels (b, d, f), *n* = 81 for NAT‐Cernusak and *n* = 33 for NE‐QLD‐Cheesman.

We compared site‐level estimates of soil N and P and leaf N and P for two transects in northern Australia, one in the Northern Territory and one in northeastern Queensland. Leaf N and P were available for these two transects, but not for the other transects in the combined dataset. These two transects provided a particularly useful comparison, because they differ in soil nutrients, specifically soil P, while experiencing similar MAT and PS (Table 1; Fig. 2). The two transects shared similar site‐level estimates for soil N (Fig. 5a); however, the estimated soil P was significantly higher for the northeastern Queensland transect compared to the Northern Territory transect (Fig. 5c). This pattern was also reflected in the ratios of soil N : P for the two transects (Fig. 5e).

Leaf N (mg g^−1^) of the sampled leaves from Northern Territory and northeastern Queensland transects did not show a significant difference (Fig. 5b), consistent with the lack of difference in estimated soil N. In the case of leaf P, however, there was a significant difference between the transects, with the northeastern Queensland transect showing higher leaf P than the Northern Territory transect (Fig. 5d), consistent with the trend for estimates of soil P. Finally, the ratio of leaf N : P was significantly higher for the Northern Territory transect compared to the northeastern Queensland transect (Fig. 5f), again consistent with estimates for soil N : P.

### Leaf Δ^13^C in relation to leaf nutrients on two transects

**Fig. 6:**
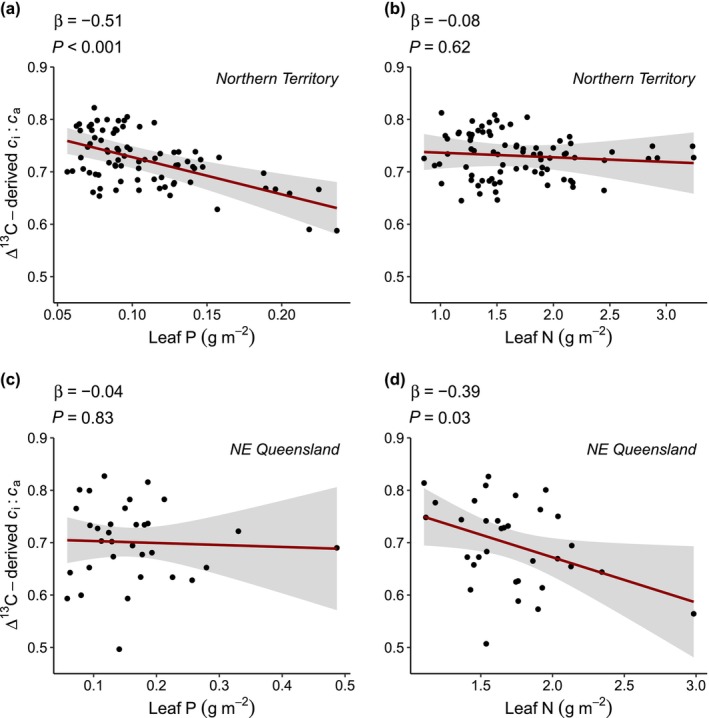
Analyses showing partial regression plots of relationships between leaf Δ^13^C–derived *c*_i_ : *c*_a_ and leaf phosphorus (a and c), and between Δ^13^C–derived *c*_i_ : *c*_a_ and leaf nitrogen (b and d). Panels (a) and (b) are based on data from the NAT‐Cernusak transect in the Northern Territory, whereas (c) and (d) use data from the NE‐QLD‐Cheesman transect in northeastern Queensland. The lines show partial linear regression fits with grey shaded areas showing their 95% confidence intervals. The standardised regression coefficient (β) for each predictor is shown at the top of the panel, along with the *P* value indicating its level of significance.

We used multiple regression analyses to understand which leaf nutrient, N or P, was more strongly associated with variation in leaf Δ^13^C on the two northern Australian transects. In the P‐impoverished soils of the Northern Territory (NAT‐Cernusak), partial regression showed a significant effect of leaf P per unit area on Δ^13^C–derived *c*_i_ : *c*_a_ (Fig. 6a). The relationship was such that Δ^13^C–derived *c*_i_ : *c*_a_ decreased with increasing leaf P, when variation in leaf N was accounted for in the model. On the other hand, there was no significant effect of leaf N per unit area on Δ^13^C–derived *c*_i_ : *c*_a_, when variation in leaf P was accounted for (Fig. 6b).

On the northeastern Queensland transect (NE‐QLD‐Cheesman), where soil P is higher, Δ^13^C‐derived *c*_i_ : *c*_a_ was not related to leaf P per unit area (Fig. 6c). Instead, there was a significant decrease in Δ^13^C‐derived *c*_i_ : *c*_a_ with increasing leaf N per unit area (Fig. 6d). The contrast between the northeastern Queensland and Northern Territory transects shows that the leaf nutrient likely to be most limiting in supply, based on soil N : P, is the one most associated with variation in leaf Δ^13^C.

### Results from two common garden experiments

**Fig. 7:**
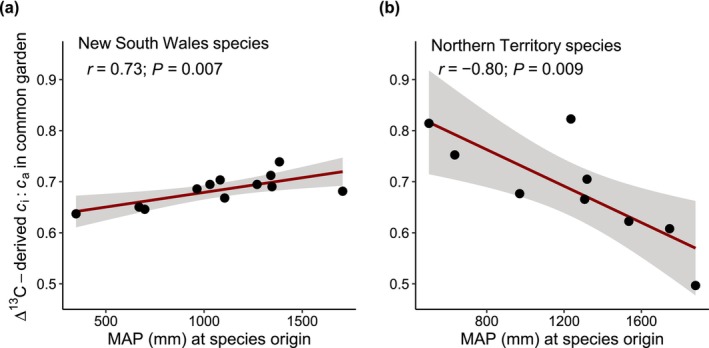
Relationships between mean annual precipitation (MAP) at the species origin and Δ^13^C–derived *c*_i_ : *c*_a_ when grown in a common garden for two different common garden experiments. Experiments were conducted with plants grown from seeds collected in (a) New South Wales in southeastern Australia and (b) the Northern Territory in northern Australia. The red lines show linear regression fits, and the grey shaded areas are their 95% confidence intervals.

We analysed data from two common garden experiments in which tree species were collected from across rainfall gradients in two regions of contrasting soil P. The regions were southeastern Australia in the state of New South Wales and northern Australia in the Northern Territory. The two sets of species showed opposite responses of leaf Δ^13^C to the MAP in the species' home ranges (Fig. 7). For the New South Wales species, there was a positive correlation between Δ^13^C‐derived *c*_i_ : *c*_a_ and MAP at the point of seed collection (Fig. 7a). By contrast, for the Northern Territory species, there was a negative correlation between Δ^13^C–derived *c*_i_ : *c*_a_ and the MAP of the species' home ranges (Fig. 7b).

### Australia‐wide leaf gas exchange dataset

**Fig. 8:**
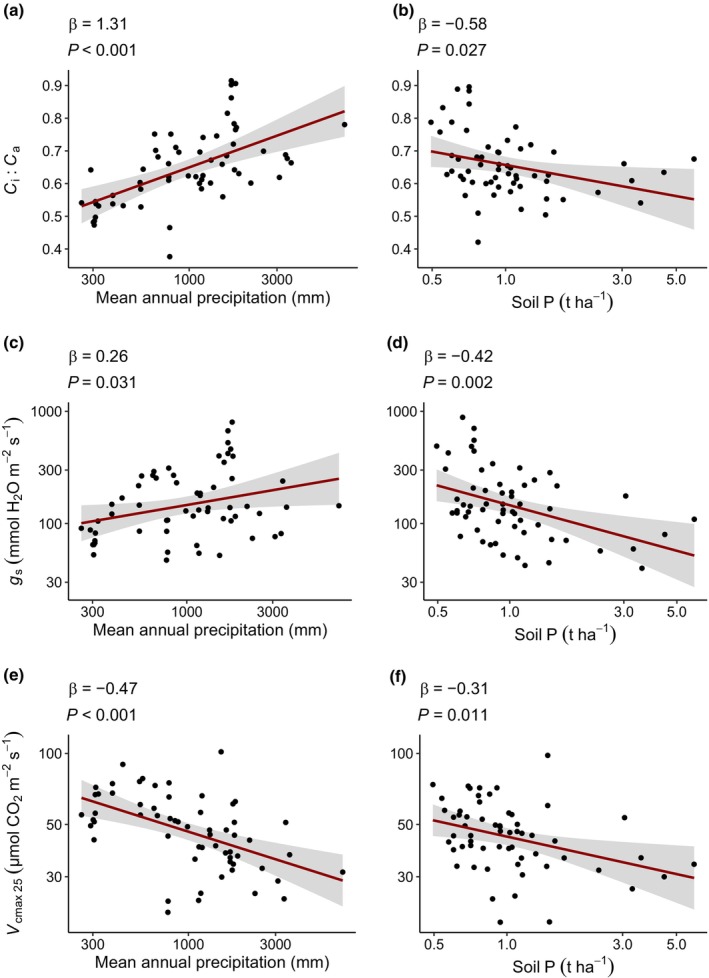
Partial regression plots showing relationships of instantaneous leaf gas exchange parameters with mean annual precipitation and soil P. Panels (a, b) show the ratio of intercellular to ambient CO_2_ concentrations, *c*_i_ : *c*_a_; panels (c, d) show stomatal conductance, *g*_s_; and panels (e, f) show the maximum carboxylation velocity of Rubisco at 25°C, *V*_cmax25_. Points represent average values at each sampling site; red lines represent partial regression lines with 95% confidence intervals shown by grey shaded areas. The standardised regression coefficient (β) for each predictor is shown at the top of the panel, along with the *P* value indicating its level of significance.

A recently compiled dataset of instantaneous measurements of leaf gas exchange in 532 species of Australian plants at 67 sites (Westerband *et al*., 2023) was used to investigate how soil P and MAP influenced *c*_i_ : *c*_a_, stomatal conductance (*g*_s_), and photosynthetic capacity, estimated as the maximum carboxylation velocity of Rubisco normalised to 25°C (*V*_cmax25_) (Fig. 8). The interaction term between MAP and soil P was not significant for any of the three response variables, and the models were therefore re‐fit without the interaction term. Partial regression analysis showed that *c*_i_ : *c*_a_ varied with both MAP and soil P, as expected based on results presented above, increasing at sites with higher MAP (Fig. 8a) and decreasing with an increase in soil P (Fig. 8b).

Stomatal conductance, *g*_s_, showed a similar trend to *c*_i_ : *c*_a_, having a positive slope with MAP (Fig. 8c) and a negative slope with soil P (Fig. 8d). The *V*_cmax25_, on the other hand, had negative relationships with both MAP (Fig. 8e) and soil P (Fig. 8f). These results indicated that Australian woody plant species at drier sites typically operate with higher *V*_cmax25_ and lower *g*_s_, in line with expectations and consistent with lower *c*_i_ : *c*_a_ at these sites. Surprisingly, both *g*_s_ and *V*_cmax25_ were lower at sites of high soil P. If the trend in *c*_i_ : *c*_a_ with soil P were driven by a relationship between soil P and photosynthetic capacity, a positive relationship between *V*_cmax_ and soil P would be expected. The fact that the relationship is negative indicates that *c*_i_ : *c*_a_ is high at sites of low soil P, not because photosynthetic capacity is low, but because *g*_s_ is high. Thus, it is variation in *g*_s_ that drives the relationship observed in Fig. 8(b), not variation in *V*_cmax25_. The results in Fig. 8 have been further visualised in three‐dimensional perspective plots in Figs S7–S9.

## Discussion

Over recent decades, several studies have reported trends in Δ^13^C with rainfall of tree communities in Australia that in combination appear to show a variable response of Δ^13^C to precipitation among regions. Here, data from these studies have been assembled, along with data from a new transect, to test whether the responses do indeed differ. After standardisation to account for changing δ^13^C of atmospheric CO_2_ over time, tissue type collected, and common sources of climate data, we found that the response of Δ^13^C to MAP does indeed vary statistically across subcontinental regions in Australia (Figs 3, S2; Table 2).

Direct measurements of soil nutrient status were not available for most of the study sites in our combined dataset. Therefore, we used the Soil and Landscapes Grid of Australia to estimate soil N and P at each site. For one of the transects in the Northern Territory (NAT‐Cernusak) and the new transect in northeastern Queensland (NE‐QLD‐Cheesman), leaf N and P concentration data were available and could be compared to the predicted trends in soil N and P. These leaf data showed that predictions of contrasting N:P ratios in the soil of the two transects were also reflected in the observed N:P ratios of the leaves (Fig. 5), supporting the idea that varying soil nutrients across the subcontinental regions play an important role in driving vegetation function. The mean value for leaf N:P on the Northern Territory transect was 16.4 g g^−1^, whereas that on the northeastern Queensland transect was 12.8 g g^−1^. It has been suggested, to a first approximation, that leaf N : P less than 14 g g^−1^ is consistent with N‐limited plant growth, whereas N : P more than 16 g g^−1^ is consistent with P‐limited plant growth (Aerts & Chapin, 2000).

With respect to leaf Δ^13^C, we showed that leaf P is a more relevant control along the Northern Territory transect (Fig. 6a,b), whereas leaf N is likely more relevant along the northeastern Queensland transect (Fig. 6c,d). This analysis showed that on the Northern Territory transect, increasing leaf P was associated with decreasing leaf Δ^13^C, or Δ^13^C–derived *c*_i_ : *c*_a_, whereas leaf N did not show a significant partial relationship with leaf Δ^13^C (Fig. 6). Leaf N, and to a lesser extent leaf P, are known to influence *c*_i_ : *c*_a_ and Δ^13^C through their relationships to leaf photosynthetic capacity (Wong *et al*., 1985). As photosynthetic capacity increases, the consumption of CO_2_ within chloroplasts increases, drawing down *c*_i_ : *c*_a_ and decreasing Δ^13^C. However, a change in *c*_i_ : *c*_a_ can also result from a change in *g*_s_, and therefore a second possibility exists to account for the observed impact of nutrients on Δ^13^C: some plants may increase transpiration as a mechanism to aid the acquisition of soil nutrients by inducing mass flow of the soil solution to root surfaces (Dalton *et al*., 1975; Fiscus, 1975; Cernusak *et al*., 2011b). In doing so, such species would have a higher *c*_i_ : *c*_a_ because of the increase in *g*_s_ required to drive higher transpiration rates. Although it was previously assumed that transpiration‐induced mass flow might not play a significant role in supplying P to root surfaces (Bieleski, 1973), more recent studies have found plant transpiration rates to be higher in plants growing at low soil P compared to plants growing in higher soil P (Garrish *et al*., 2010; Maire *et al*., 2015; Huang *et al*., 2017). This supports the idea that plants may use transpiration‐induced mass flow of the soil solution as a mechanism of P foraging (Cernusak *et al*., 2010; Garrish *et al*., 2010).

To examine whether changes in photosynthetic capacity or in *g*_s_ are more likely to drive a relationship between Δ^13^C and soil P across Australia, we took advantage of a recently compiled, continent‐wide dataset of leaf photosynthetic characteristics (Westerband *et al*., 2023). With this dataset, we tested whether the mechanism of soil P influencing Δ^13^C more likely results from increased photosynthetic capacity drawing down *c*_i_ : *c*_a_, or alternatively, whether it more likely relates to water‐use strategies that would favour high *g*_s_ and thus high *c*_i_ : *c*_a_ in low P soils. That is, assuming *c*_i_ : *c*_a_ responds to soil P in the Australia‐wide dataset, is the response of decreasing *c*_i_ : *c*_a_ with increasing soil P driven by changing *g*_s_ or photosynthetic capacity (*V*_cmax25_)? Using multiple regression with MAP and soil P as independent variables, we observed that *c*_i_ : *c*_a_ varied as expected with each of these, increasing with increasing MAP and decreasing with increasing soil P (Fig. 8a,b). We then tested *g*_s_, which also increased with increasing MAP, as expected (Fig. 8c). Importantly, however, *g*_s_ also decreased with increasing soil P (Fig. 8d), signalling that stomatal conductance could be important in modulating the response of *c*_i_ : *c*_a_, and therefore Δ^13^C, to soil P. Finally, we observed that *V*_cmax25_ showed a negative relationship with soil P (Fig. 8f). This is opposite to what would be expected if soil P modulated *c*_i_ : *c*_a_ through a positive relationship with photosynthetic capacity. Instead, the implication is that low P soils in Australia favour plants which operate with higher *g*_s_, which in turn results in higher *c*_i_ : *c*_a_, higher Δ^13^C, and lower WUE_i_.

Strategies in Australian trees for liberating P in P‐impoverished soils include the release of carboxylates and extracellular phosphatase enzymes into the rhizosphere, among other strategies (Lambers *et al*., 2021; Shen, Ranathunge, de Tombeur, *et al*., 2024; Zhou *et al*., 2024). Carboxylates lower the pH in the vicinity of roots, allowing solubilisation of otherwise inaccessible soil P, while phosphatase enzymes hydrolyse phosphate, which can be absorbed by roots, from organic P‐containing molecules that cannot otherwise be absorbed. Both carboxylates and extracellular phosphatase enzymes in this context involve the release of resources into the rhizosphere to liberate P, which is then available not just to the plant that expended the resources, but also to neighbouring competitors. If the liberated P is captured by a competitor, or lost to leaching or runoff, it represents a wasted investment on the part of the resource‐releasing plant. Evidence of this is provided by observations of so‐called facilitation of P‐acquisition by non‐P‐mining plants that are neighbours to P‐mining plants (Shen, Ranathunge, Zhong, *et al*., 2024, Shen, Ranathunge, de Tombeur, *et al*., 2024). A complementary strategy would be for the P‐mining plant to use high transpiration rates to collect liberated P within the rhizosphere, especially where a plant has invested heavily in cleaving phosphate from recalcitrant sources. In fact, it has been observed in southwestern Australia that eucalypts and other tree species that invest heavily in exudation of carboxylates also pair this strategy with low WUE_i_, manifested as a larger leaf Δ^13^C (Shen, Ranathunge, de Tombeur, *et al*., 2024).

Such strategies may extend beyond Australia, as a negative correlation between soil P availability and *g*_s_ has also been observed at the global scale (Maire *et al*., 2015). The diel cycle of transpiration may in fact provide the opportunity for a nightly pulse of P‐mining compounds to be released and diffuse away from root surfaces, followed by a daily collection of the proceeds as transpiration drives movement of the soil solution back to those same root surfaces. Eucalypt roots are also known to associate with both arbuscular and ecto‐mycorrhizas (Ashton, 1976; Newman & Reddell, 1987; Wang & Qiu, 2006), which may additionally play a role in release of carboxylates into the rhizosphere (Sardans *et al*., 2023; Zhang *et al*., 2023).

Two common garden studies provided further insight into our investigation of the interaction between soil P and MAP in modulating Δ^13^C (Anderson *et al*., 1996; Cernusak, 2020). The two common garden studies were fortuitously situated, with one using southeastern Australian tree species and the other using Northern Territory tree species. The two studies showed that when grown in common conditions, the two sets of species had contrasting relationships between Δ^13^C–derived *c*_i_ : *c*_a_ and MAP either at their point of seed collection, in the case of the southeastern Australian species, or for the species' home ranges, in the case of the Northern Territory species (Fig. 7). It is also apparent that although Δ^13^C of the southeastern Australian species increased significantly in the common garden with increasing MAP at the site of origin, the overall variability among species was less than that in the Northern Territory species (Fig. 7). This can be compared with recently reported results for a study in Victoria in which four common gardens were established with 10 eucalypt species that occur along the rainfall gradient related to the VIC‐Givnish transect (Smith *et al*., 2023). In this case, no significant effect was observed of the ratio of MAP to pan evaporation in the species' home ranges on the Δ^13^C of the species within the common gardens. Thus, the more muted response in Fig. 7(a) for the common garden in New South Wales is consistent with the Victorian results.

The contrasting relationships between Δ^13^C and MAP at the site of origin for the species shown in Fig. 7 provide two important insights. The first is that the different Δ^13^C‐MAP slopes in the Northern Territory compared to southeastern Australia are likely associated with species replacements that tend to reinforce this divergence (Schulze *et al*., 2006b). In the Northern Territory, as species are replaced along the transect from high rainfall to low rainfall, this likely tempers the change in Δ^13^C because the drier zone species replacing the wetter zone species show a higher Δ^13^C, at least in well‐watered conditions. On the other hand, in southeastern Australia, the drier zone species replacing the wetter zone species will tend to have lower Δ^13^C, all else being equal. The second important insight is that the environmental conditions in these two regions appear to have selected for different tree behaviours in relation to variation in MAP. Through evolutionary time, our analysis suggests that one of the selecting forces was the severe P impoverishment of the soils in the Northern Territory. There, drier zone species evolved to have low WUE_i_, we presume to facilitate P foraging and photosynthetic P‐use efficiency, causing a lesser increase in WUE_i_ in response to declining MAP along the transect.

Previous research has established that an important aspect of the relationship between Δ^13^C and MAP in eucalypts in Australia is the coordinated response of SLA (Schulze *et al*., 2006a, 2014; Turner *et al*., 2008). Our results are consistent with this, insofar as we observed general increases in SLA with increasing MAP across the transects (Fig. S4), and a general relationship between Δ^13^C and SLA, or its inverse LMA (Fig. S5). In further analysis, we observed that the relationship between Δ^13^C and LMA interacted significantly with soil P, such that at low soil P, there was a weaker relationship between Δ^13^C and LMA than at higher soil P (Fig. S6). Bringing this together with the discussion above, we suggest that transects in regions with lower soil P, such as in the Northern Territory and in southwestern Australia, will present lower SLA in the wetter parts of the transects and therefore the Δ^13^C in the wetter parts will also reach lower maximum values, consistent with trends presented in Figs S2 and 3 for Δ^13^C–derived *c*_i_ : *c*_a_. Then, moving from the wetter to drier parts of these transects, the decrease in Δ^13^C will be less in low P regions than in high P regions because lower WUE_i_ will be favoured for purposes of P foraging. That would then result in the Δ^13^C‐MAP slopes of transects in low P regions being less than those in high P regions.

Vast parts of the Australian landscape have been severely impoverished in soil P over timescales sufficiently long to drive evolution that favours P foraging in P‐depleted soils, even in the face of other resource constraints such as water availability. In analyses presented here, we have shown that across multiple datasets, the well‐known association between *c*_i_ : *c*_a_ and MAP, inferred from leaf Δ^13^C or measured instantaneously at the leaf level, differs according to regional soil P across the Australian continent. In P‐poor, drier regions, species have been selected for which have lower WUE_i_ and which increase their WUE_i_ to a lesser extent when exposed to lower MAP. The low WUE_i_ is caused by higher *g*_s_. This benefits these plants in at least two ways: it raises the intercellular CO_2_ concentration, increasing their photosynthetic rates, all else being equal, and it draws more of the soil solution to root surfaces, increasing nutrient foraging and the capture of P liberated from the soil through exudation of carboxylates and phosphatases. While these strategies may also occur on other continents, we expect them to be especially pronounced within the Australian flora due to the geological and biogeographical histories of the continent.

## Competing interests

None declared.

## Author contributions

IA, AWC, and LAC collected new data, conducted statistical analyses, and drafted the first version of the manuscript. GDF, TJG, MGDK, E‐DS, ACW, and IJW suggested additional analyses and interpretation and contributed to editing the final version of the manuscript.

## Disclaimer

The New Phytologist Foundation remains neutral with regard to jurisdictional claims in maps and in any institutional affiliations.

## Supporting information


**Fig. S1** A pairwise scatterplot matrix of the climatic and soil variables taken as candidate predictors of carbon isotope discrimination.
**Fig. S2** A figure similar to Fig. 3 of the main text, but here showing the relationship between carbon isotope discrimination and mean annual precipitation for the studied transects.
**Fig. S3** A three‐dimensional perspective plot showing the predictions of a multiple regression model in which carbon isotope discrimination was the response variable and mean annual precipitation, soil P, and their interaction were independent variables.
**Fig. S4** Relationships between site‐averaged specific leaf area and mean annual precipitation for transects included in our analysis.
**Fig. S5** Carbon isotope discrimination plotted as a function of leaf mass per unit area.
**Fig. S6** A scatterplot showing observations of carbon isotope discrimination as a function of leaf mass per area, overlain with predictions from a multiple regression model in which carbon isotope discrimination was fitted as a function of leaf mass per area, soil P, and their interaction.
**Fig. S7** A three‐dimensional perspective plot showing a model in which the ratio of intercellular to ambient CO_2_ concentrations was predicted as a function of mean annual precipitation and soil P.
**Fig. S8** A three‐dimensional perspective plot showing predictions of the natural logarithm of stomatal conductance as a function of mean annual precipitation and soil P.
**Fig. S9** A three‐dimensional perspective plot showing predictions of the natural logarithm of maximum carboxylation velocity of Rubisco normalised to 25°C as a function of mean annual precipitation and soil P.
**Table S1** Tree species sampled at each site included in analyses of transect data.
Please note: Wiley is not responsible for the content or functionality of any Supporting Information supplied by the authors. Any queries (other than missing material) should be directed to the *New Phytologist* Central Office.

## Data Availability

All data and code for the analyses presented in this paper are publicly available in a Dryad data repository doi: 10.5061/dryad.zgmsbccst.
