# Supplementary material for: Soil phosphorus drives subcontinental patterns of carbon isotope discrimination across Australia
Source: New Phytol. 2026 Mar 19;251(3):1038–53. doi: 10.1111/nph.71069 (PMC13326502; doi:10.1111/nph.71069)
Supplement: Supplementary file 1 — Fig. S1 A pairwise scatterplot matrix of the climatic and soil variables taken as candidate predictors of carbon isotope discrimination. Fig. S2 A figure similar to Fig. 3 of the main text, but here showing the relationship between carbon isotope discrimination and mean annual precipitation for the studied transects. Fig. S3 A three‐dimensional perspective plot showing the predictions of a multiple regression model in which carbon isotope discrimination was the response variable and mean annual precipitation, soil P, and their interaction were independent variables. Fig. S4 Relationships between site‐averaged specific leaf area and mean annual precipitation for transects included in our analysis. Fig. S5 Carbon isotope discrimination plotted as a function of leaf mass per unit area. Fig. S6 A scatterplot showing observations of carbon isotope discrimination as a function of leaf mass per area, overlain with predictions from a multiple regression model in which carbon isotope discrimination was fitted as a function of leaf mass per area, soil P, and their interaction. Fig. S7 A three‐dimensional perspective plot showing a model in which the ratio of intercellular to ambient CO2 concentrations was predicted as a function of mean annual precipitation and soil P. Fig. S8 A three‐dimensional perspective plot showing predictions of the natural logarithm of stomatal conductance as a function of mean annual precipitation and soil P. Fig. S9 A three‐dimensional perspective plot showing predictions of the natural logarithm of maximum carboxylation velocity of Rubisco normalised to 25°C as a function of mean annual precipitation and soil P. Table S1 Tree species sampled at each site included in analyses of transect data. Please note: Wiley is not responsible for the content or functionality of any Supporting Information supplied by the authors. Any queries (other than missing material) should be directed to the New Phytologist Central Office. [file NPH-251-1038-s001.docx]

***New Phytologist* Supporting Information**

Article title: Soil phosphorus drives subcontinental patterns of carbon-isotope discrimination across Australia

Authors: Iftakharul Alam, Alexander W. Cheesman, Graham D. Farquhar, Thomas J. Givnish, Martin G. De Kauwe, Ernst-Detlef Schulze, Andrea C. Westerband, Ian J. Wright, and Lucas A. Cernusak

Article acceptance date: 01 February 2026

The following Supporting Information is available for this article:

**Fig. S1** A pairwise scatterplot matrix of the climatic and soil variables taken as candidate predictors of carbon isotope discrimination.

**Fig. S2** A figure similar to Figure 3 of the main text, but here showing the relationship between carbon isotope discrimination and mean annual precipitation for the studied transects.

**Fig. S3** A three-dimensional perspective plot showing the predictions of a multiple regression model in which carbon isotope discrimination was the response variable and mean annual precipitation, soil P, and their interaction were independent variables.

**Fig. S4** Relationships between site-averaged specific leaf area and mean annual precipitation for transects included in our analysis.

**Fig. S5** Carbon isotope discrimination plotted as a function of leaf mass per unit area.

**Fig. S6** A scatterplot showing observations of carbon isotope discrimination as a function of leaf mass per area, overlain with predictions from a multiple regression model in which carbon isotope discrimination was fitted as a function of leaf mass per area, soil P, and their interaction.

**Fig. S7** A three-dimensional perspective plot showing a model in which the ratio of intercellular to ambient CO_2_ concentrations was predicted as a function of mean annual precipitation and soil P.

**Fig. S8** A three-dimensional perspective plot showing predictions of the natural logarithm of stomatal conductance as a function of mean annual precipitation and soil P.

**Fig. S9** A three-dimensional perspective plot showing predictions of the natural logarithm of maximum carboxylation velocity of Rubisco normalised to 25°C as a function of mean annual precipitation and soil P.

**Table S1** Tree species sampled at each site included in analyses of transect data.

**Fig. S1**

**
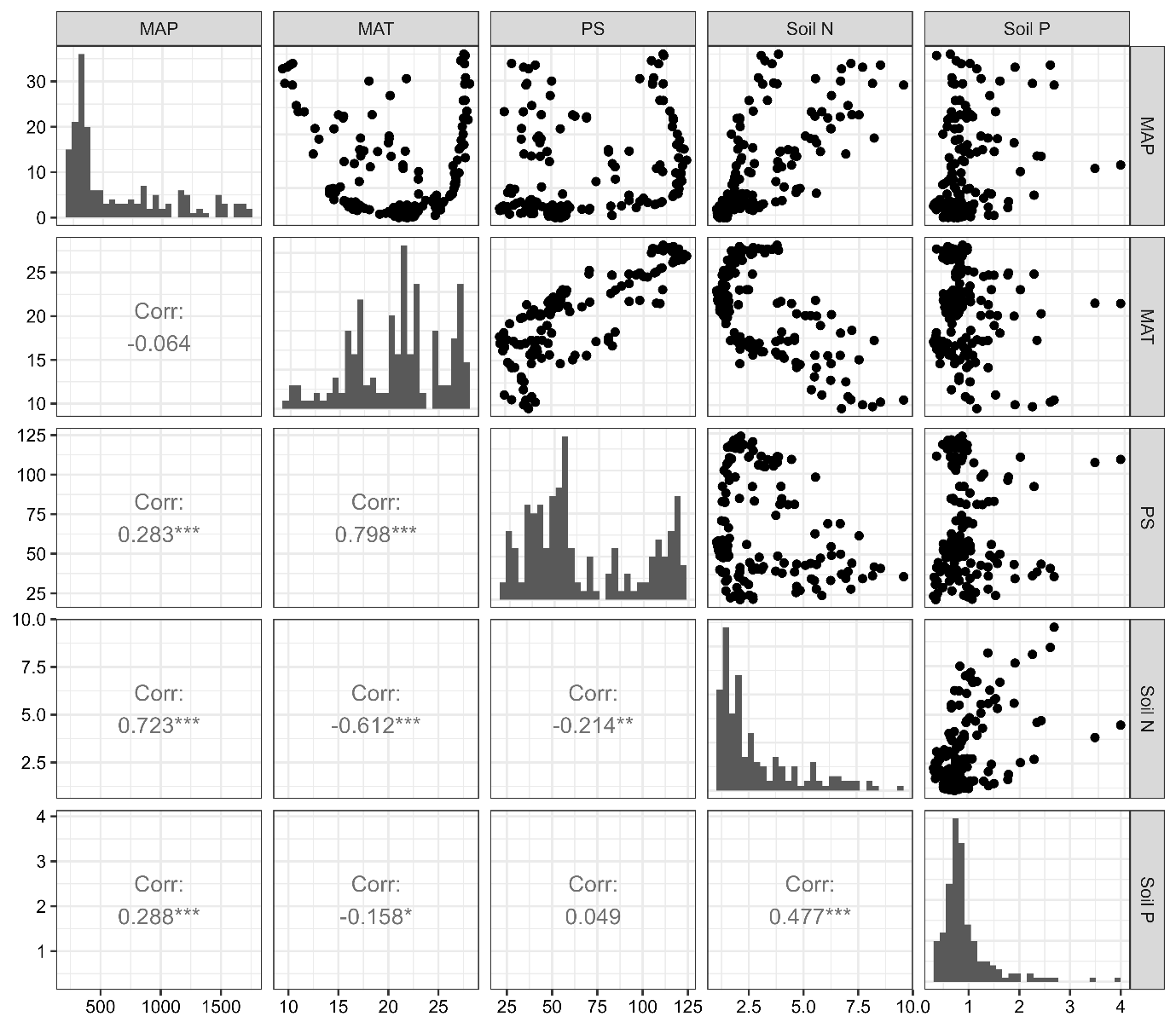
**

**Fig. S1.** A pairwise scatterplot matrix of the climatic and soil variables taken as candidate predictors of carbon isotope discrimination (Δ^13^C; ‰): MAP is mean annual precipitation (mm); MAT is mean annual temperature (°C); PS is precipitation seasonality (%); soil N is soil nitrogen concentration (t ha^-1^); and soil P is soil phosphorus concentration (t ha^-1^). Scatterplots are shown above the diagonal, histograms along the diagonal, and Pearson correlation coefficients with significance levels below the diagonal (****p* < 0.001; ***p* < 0.01; **p* < 0.05).

**Fig. S2**


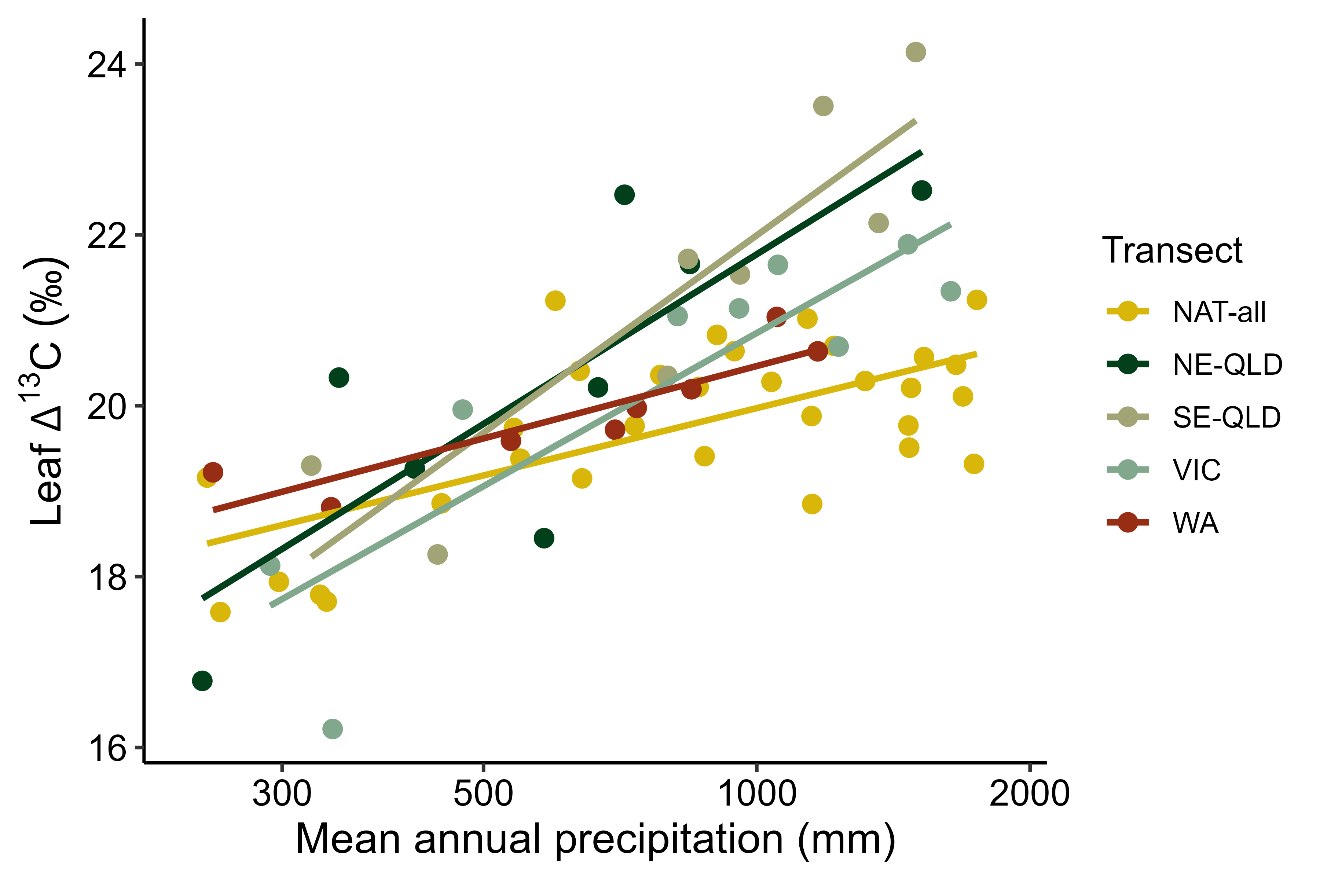


**Fig. S2.** A figure similar to Figure 3 of the main text, but here showing the relationship between leaf Δ^13^C and mean annual precipitation for the studied transects, whereas Figure 3 of the main text shows the model-derived construct, Δ^13^C–derived *c*_i_/*c*_­a_. Calculating Δ^13^C–derived *c*_i_/*c*_­a_ involves a linear transformation of Δ^13^C, following Equation 1 of the main text. The solid lines show modelled regression fits for each transect. The three sources of data for the NAT transect were included in the model as a random effect. The fixed model effects are shown in the figure, and the x-axis is logarithmic (natural logarithm).

**Fig. S3**


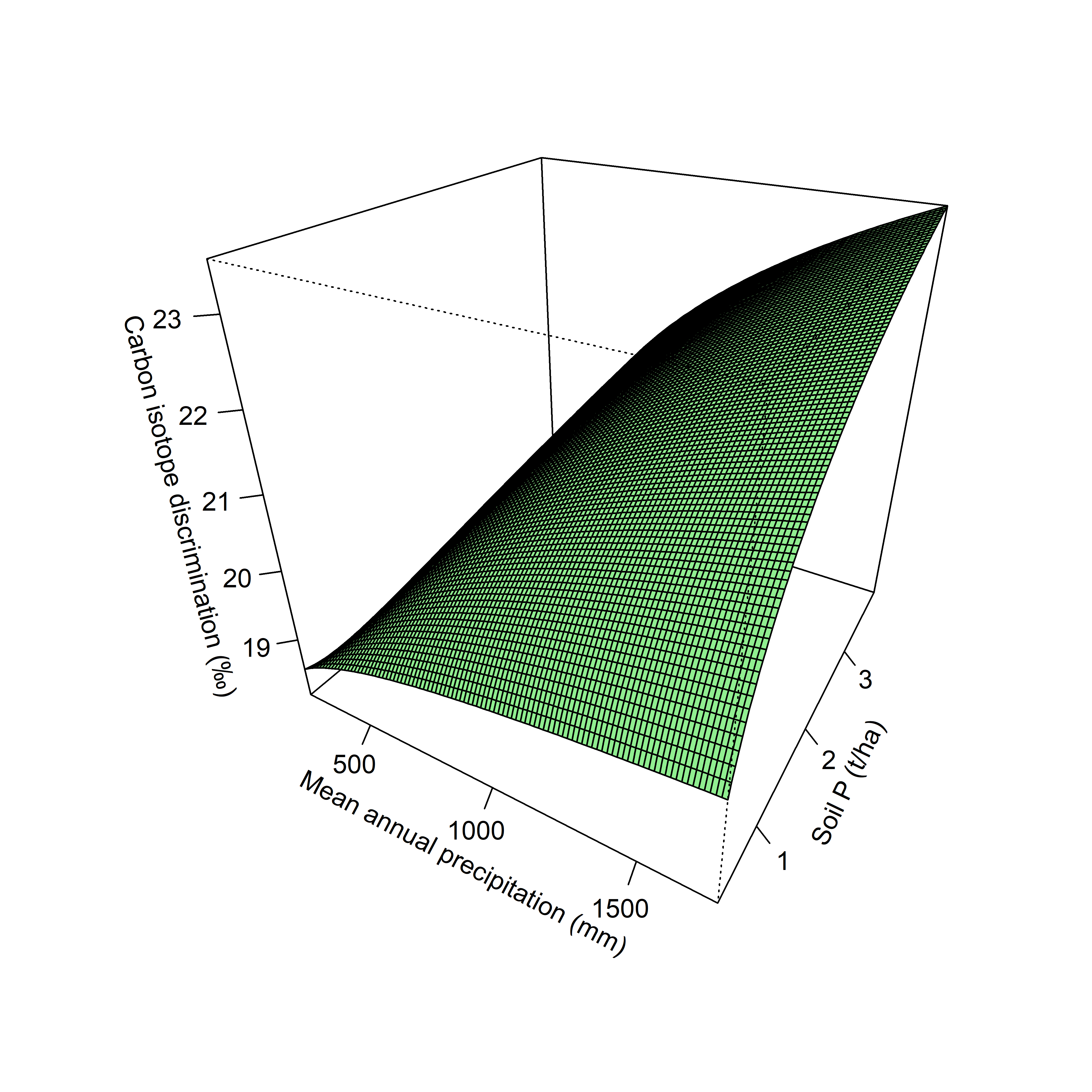


**Fig. S3.** A three-dimensional perspective plot showing the predictions of a multiple regression model in which carbon isotope discrimination (Δ^13^C; ‰) was the response variable and mean annual precipitation (MAP; mm), soil P (t ha^-1^), and their interaction were independent variables. Model results are shown in the summary table below. The regression model shows a much stronger response of Δ^13^C to MAP at high compared to low soil P, and that at low MAP, the response of Δ^13^C to soil P is such that Δ^13^C increases as soil P decreases. The adjusted *R*^2^ for the model is 0.44, with *n* = 174 sites.

|  | **Carbon isotope discrimination (Δ^13^C; ‰)** | | |
| --- | --- | --- | --- |
| *Predictors* | *Estimates* | *CI* | *p* |
| (Intercept) | 10.65 | 8.76 – 12.55 | **<0.001** |
| ln(MAP) | 1.42 | 1.13 – 1.72 | **<0.001** |
| ln(Soil P) | -4.97 | -9.27 – -0.66 | **0.024** |
| ln(MAP) × ln(Soil P) | 0.88 | 0.21 – 1.56 | **0.010** |

**Fig. S4**


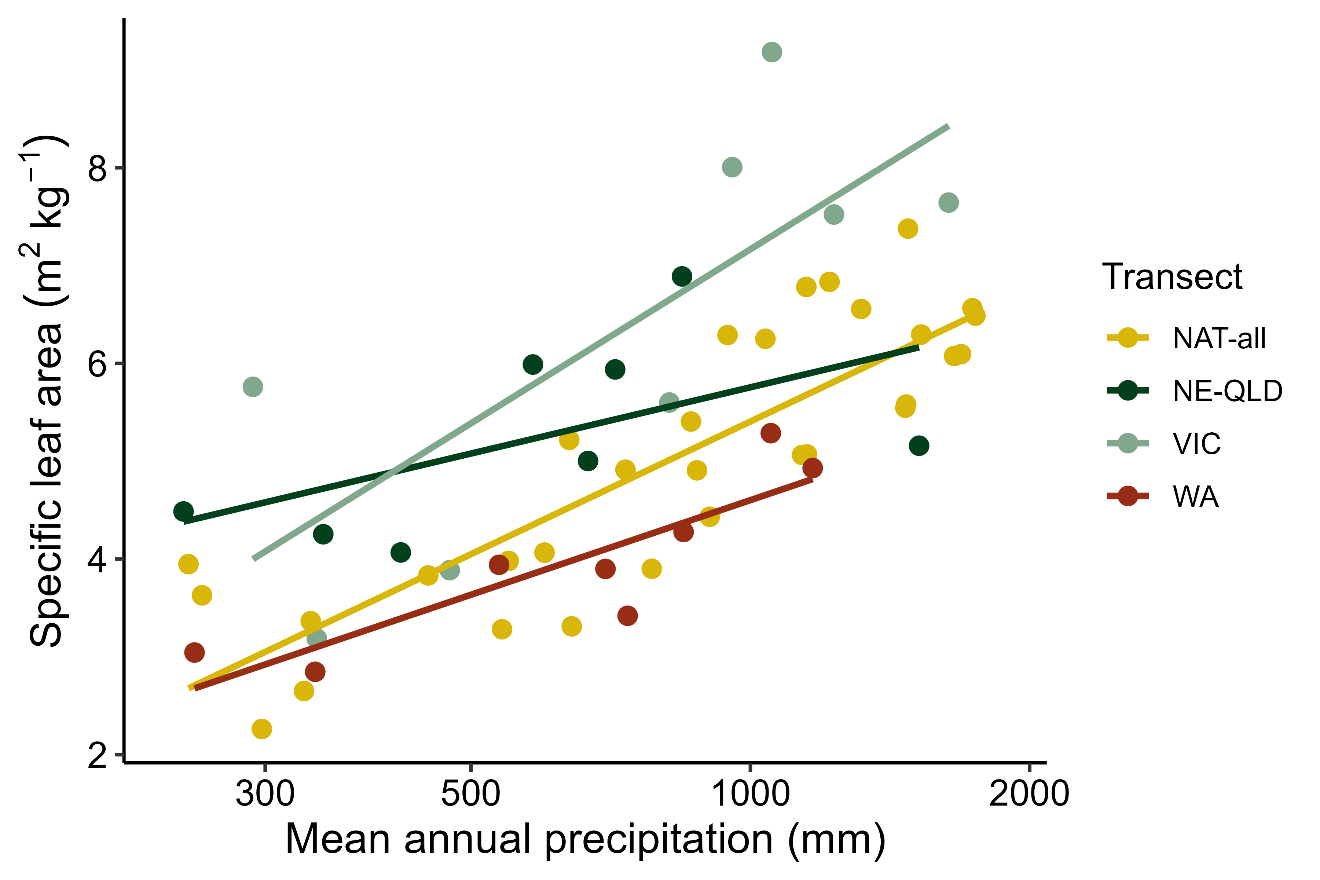


**Fig. S4.** Relationships between site-averaged specific leaf area (SLA) and mean annual precipitation (MAP) for transects included in our analysis. Note that the transect SE-QLD-Stewart is not shown because specific leaf area data were not available for this transect. The solid lines show modelled regression fits for each transect predicted by a linear mixed-effects model. The three sources of data for the NAT transect were included in the model as a random effect. The fixed model effects are shown in the figure. The x-axis is shown on a logarithmic scale (natural logarithm).

**Fig. S5**


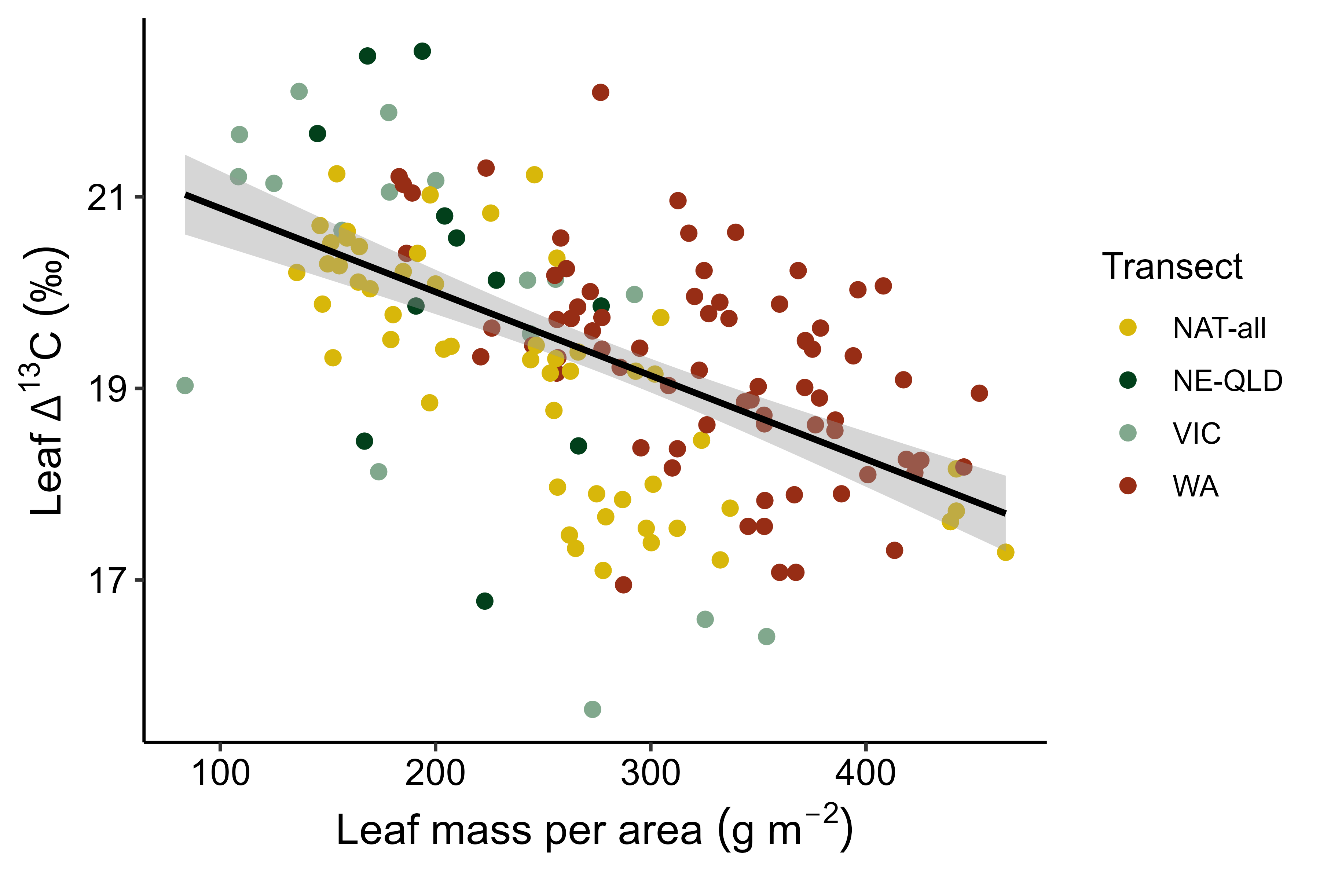


**Fig. S5.** Carbon isotope discrimination (Δ^13^C) plotted as a function of leaf mass per unit area (LMA), which is the inverse of specific leaf area. Note that the transect SE-QLD-Stewart is not shown because specific leaf area data were not available for this transect. The black line shows a linear regression fit to all data. Points represent site averages across the full data set. The grey shading shows the 95% confidence interval of the regression fit. The regression fit is described by the equation Δ^13^C = -0.0087LMA + 21.75 (*R*^2^ = 0.34, *F*_(1, 156)_ = 80.09, *p* < .001).

**Fig. S6**


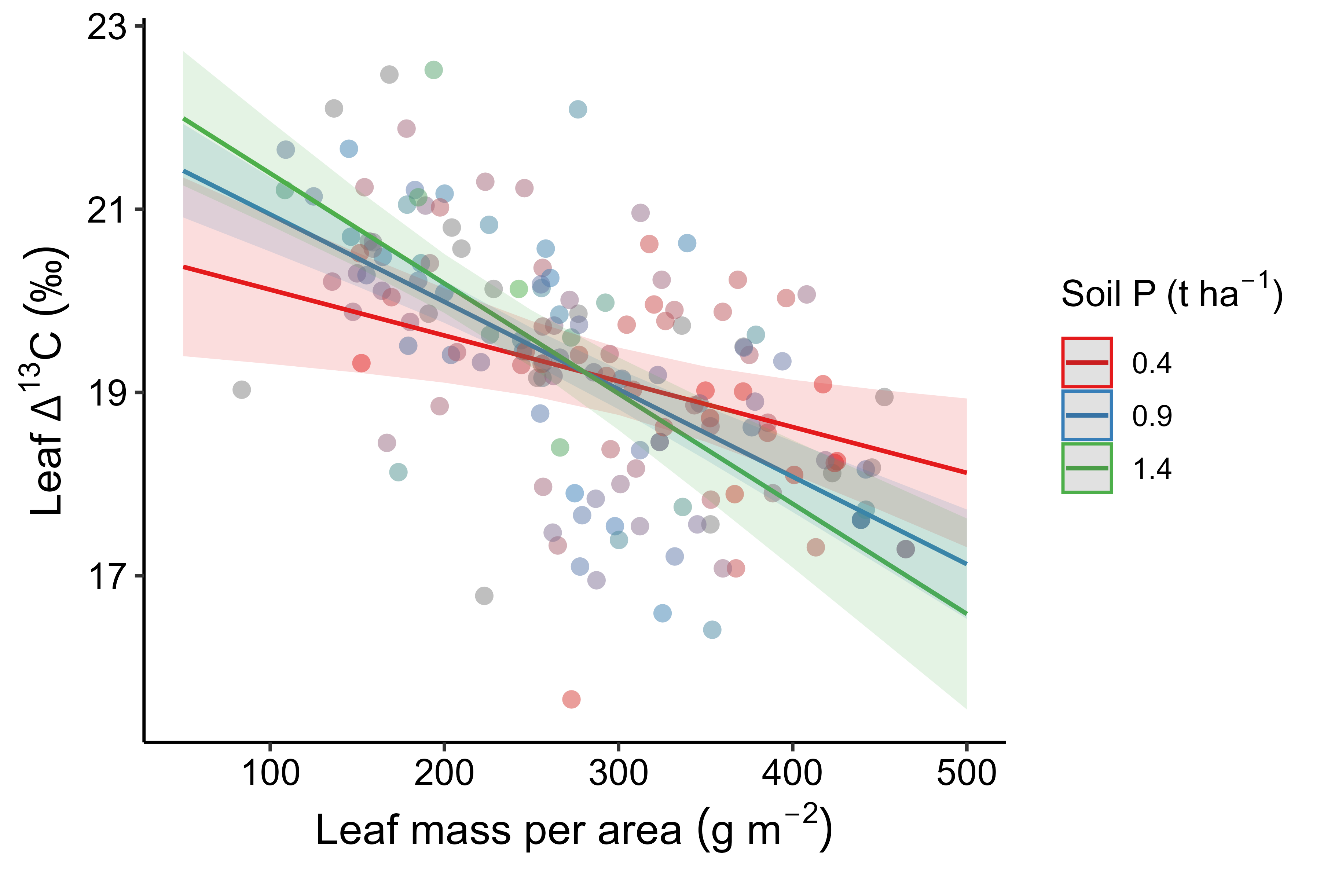


**Fig. S6.** A scatterplot showing observations of carbon isotope discrimination (Δ^13^C; ‰) as a function of leaf mass per area (LMA), overlain with predictions from a multiple regression model in which Δ^13^C was fitted as a function of LMA, soil P (t ha^-1^), and their interaction. Predictions are shown for three levels of soil P, approximating the mean soil P ± 1 SD. Points in the scatterplot are coloured according to the level of soil P for each observation. The regression model summary results are shown below. The model shows that at low soil P, Δ^13^C is a weaker function of LMA than at high soil P. The adjusted *R*^2^ for the model is 0.35, with *n* = 158 sites.

|  | **Carbon isotope discrimination (Δ^13^C; ‰)** | | |
| --- | --- | --- | --- |
| *Predictors* | *Estimates* | *CI* | *p* |
| (Intercept) | 22.06 | 21.41 – 22.72 | **<0.001** |
| LMA | -0.01 | -0.01 – -0.01 | **<0.001** |
| ln(Soil P) | 1.57 | 0.27 – 2.88 | **0.018** |
| LMA × ln(Soil P) | -0.01 | -0.01 – -0.00 | **0.021** |

**Fig. S7**


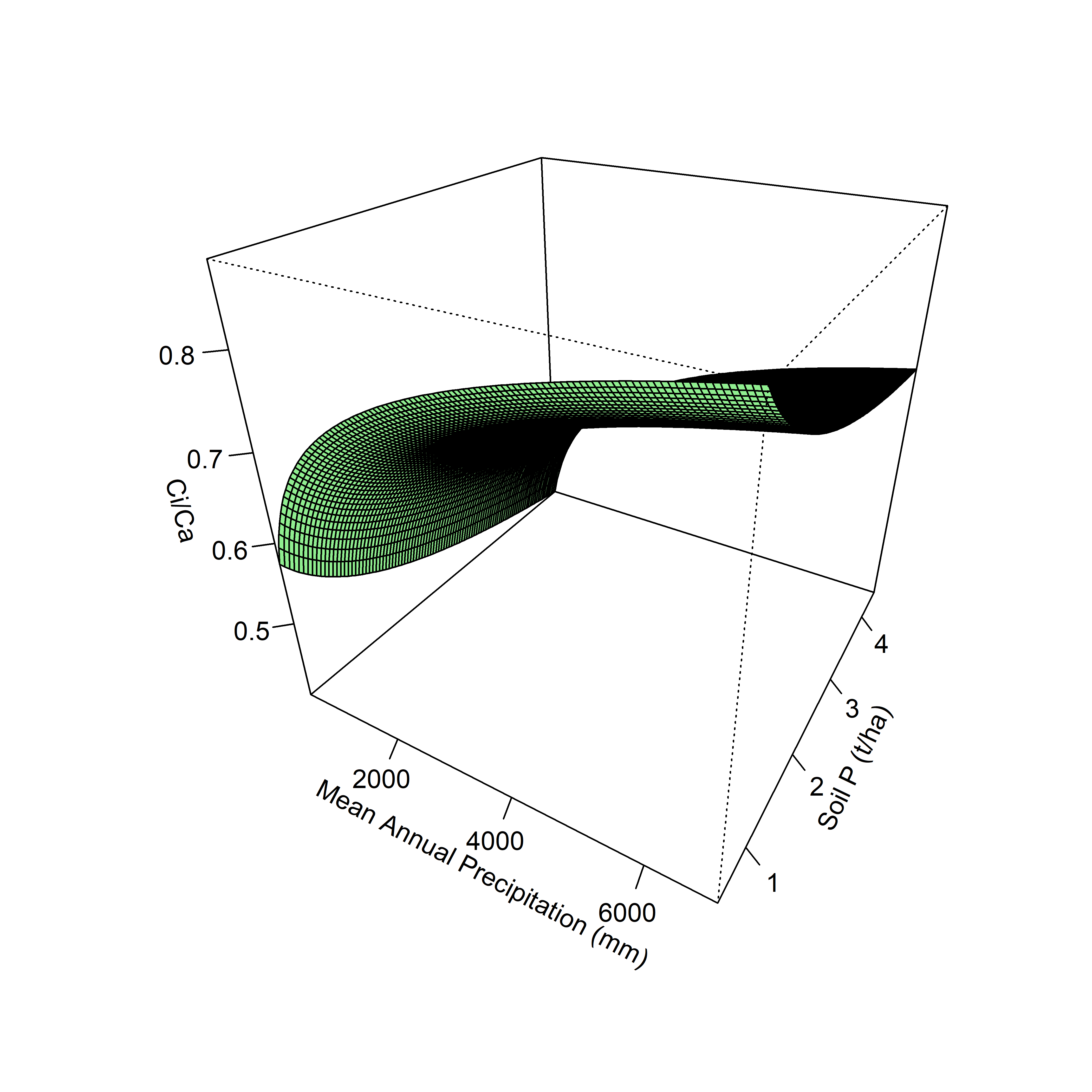


**Fig. S7.** A three-dimensional perspective plot showing a model in which the ratio of intercellular to ambient CO_2_ concentrations, *c*_i_/*c*_­a_, was predicted as a function of mean annual precipitation and soil P. The dataset employed is from Westerband *et al*. (2023; citation provided in main article) and comprises instantaneous gas exchange measurements for woody plants across Australia. Regression results are shown below and are further visualised in Figures 8a and 8b of the main text. The interaction between ln(MAP) and ln(soil P) was not significant, and the model was therefore re-fitted without an interaction term. The adjusted *R*^2^ for the model is 0.27, with *n* = 59 sites.

|  | ***c*_i_/*c*_a_** | | |
| --- | --- | --- | --- |
| *Predictors* | *Estimates* | *CI* | *p* |
| (Intercept) | 0.04 | -0.21 – 0.29 | 0.775 |
| ln(MAP) | 0.09 | 0.05 – 0.12 | **<0.001** |
| ln(Soil P) | -0.06 | -0.11 – -0.01 | **0.027** |

**Fig. S8**


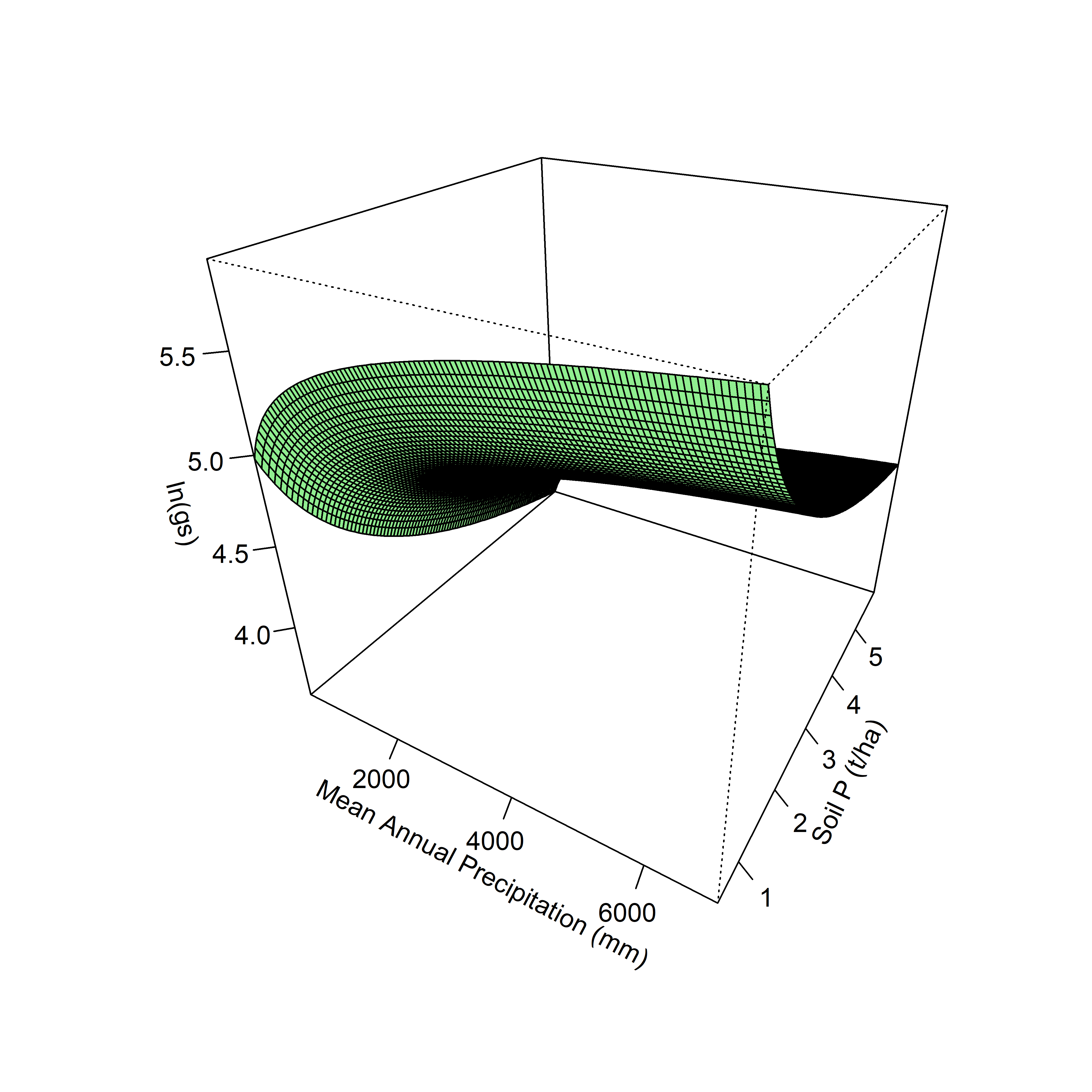


**Fig. S8.** A three-dimensional perspective plot showing predictions of the natural logarithm of stomatal conductance, ln(*g*_s_) (mmol m^2^ s^-1^), as a function of mean annual precipitation and soil P. The dataset employed is the same as that for Figure S6. Regression results are shown below and are further visualised in Figures 8c and 8d of the main text. The interaction between ln(MAP) and ln(soil P) was not significant, and the model was therefore re-fitted without the interaction term. The adjusted *R*^2^ for the model is 0.13, with *n* = 60 sites.

|  | **ln(*g*_s_)** | | |
| --- | --- | --- | --- |
| *Predictors* | *Estimates* | *CI* | *p* |
| (Intercept) | 3.03 | 1.29 – 4.77 | **0.001** |
| ln(MAP) | 0.28 | 0.03 – 0.53 | **0.031** |
| ln(Soil P) | -0.58 | -0.94 – -0.22 | **0.002** |

**Fig. S9**


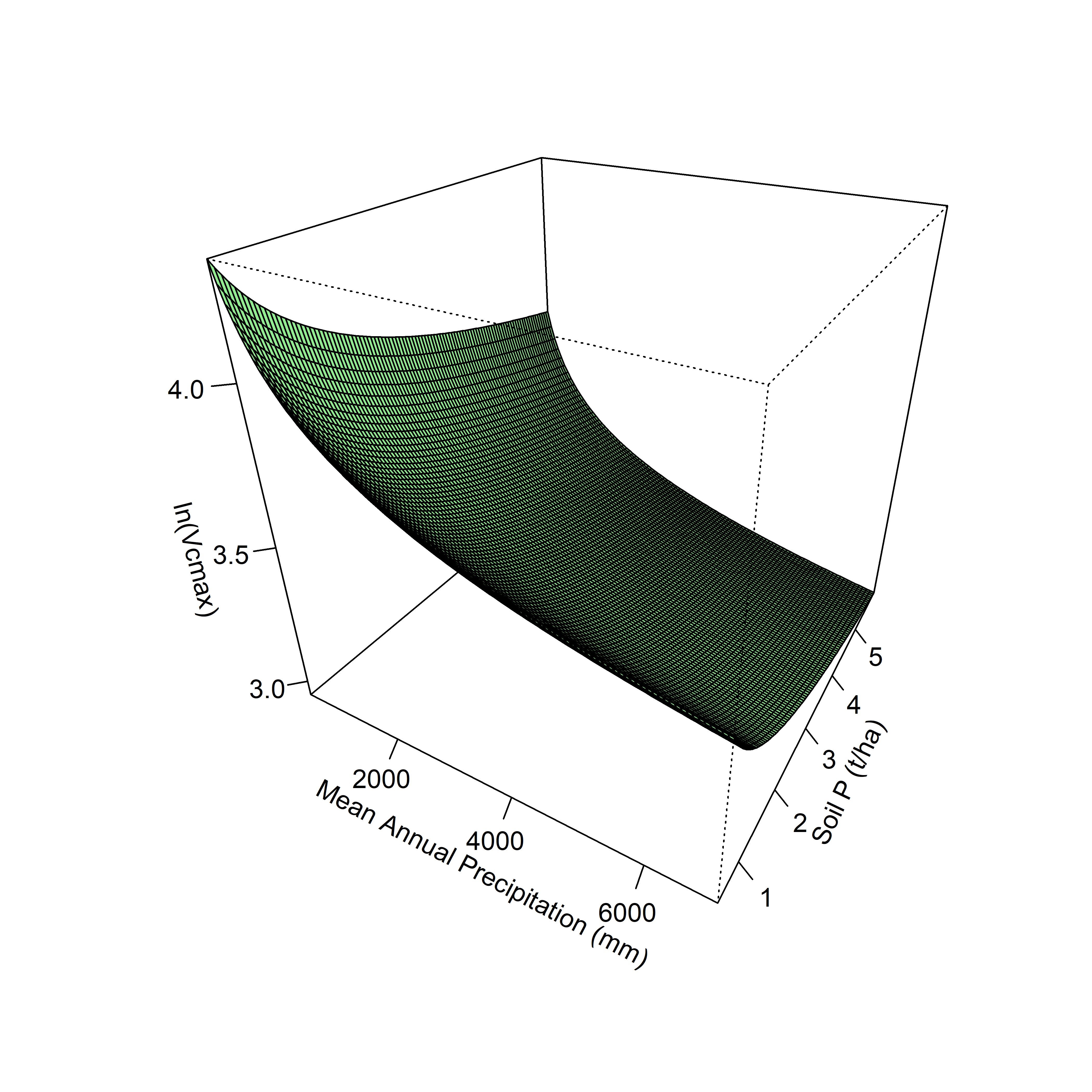


**Fig. S9.** A three-dimensional perspective plot showing predictions of the natural logarithm of maximum carboxylation velocity of Rubisco normalised to 25°C, ln(*V*_cmax25_) (µmol CO_2_ m^2^ s^-1^), as a function of mean annual precipitation and soil P. The dataset employed is the same as that for Figure S6. Regression results are shown below and are further visualised in Figures 8e and 8f of the main text. The interaction between ln(MAP) and ln(soil P) was not significant, and the model was therefore re-fitted without the interaction term. The adjusted *R*^2^ for the model is 0.40, with *n* = 60 sites.

|  | **log(*V*_cmax25_)** | | |
| --- | --- | --- | --- |
| *Predictors* | *Estimates* | *CI* | *p* |
| (Intercept) | 5.52 | 4.69 – 6.35 | **<0.001** |
| ln(MAP) | -0.25 | -0.36 – -0.13 | **<0.001** |
| ln(Soil P) | -0.23 | -0.40 – -0.05 | **0.011** |

**Table S1.** Tree species sampled at each site included in analyses of transect data.

| Site Id number | Site Code | Latitude | Longitude | Transect-Source | Species |
| --- | --- | --- | --- | --- | --- |
| 1 | STN_1 | -20.7148 | 145.2249 | NE-QLD-Cheesman | *Eucalyptus crebra* F. Muell |
| 2 | STN_2 | -21.0635 | 144.0161 | NE-QLD-Cheesman | *Eucalyptus* sp. |
| 3 | STN_3 | -22.2297 | 142.4081 | NE-QLD-Cheesman | *Corymbia dallachiana* (Benth.) K.D. Hill & L.A.S. Johnson |
| 4 | STN_4 | -22.2211 | 142.1022 | NE-QLD-Cheesman | *Corymbia erythrophloia* (Blakely) K.D. Hill & L.A.S. Johnson |
| 5 | STN_5 | -22.5511 | 141.2857 | NE-QLD-Cheesman | *Eucalyptus crebra* F. Muell |
| 6 | STN_6 | -23.073 | 139.9495 | NE-QLD-Cheesman | *Corymbia trachyphloia* (F. Muell.) K.D. Hill & L.A.S. Johnson |
| 7 | STN_7 | -18.8811 | 144.5336 | NE-QLD-Cheesman | *Eucalyptus coolabah* Blakely & Jacobs Blakely & Jacobs |
| 8 | STN_8 | -18.1537 | 144.7597 | NE-QLD-Cheesman | *Eucalyptus coolabah* Blakely & Jacobs Blakely & Jacobs |
| 9 | STN_9 | -18.0926 | 144.844 | NE-QLD-Cheesman | *Eucalyptus normantonensis* Maiden & Cambage |
| 10 | STN_10 | -17.6484 | 145.2815 | NE-QLD-Cheesman | *Eucalyptus coolabah* Blakely & Jacobs Blakely & Jacobs |
| 11 | STN_11 | -16.9937 | 145.5672 | NE-QLD-Cheesman | *Eucalyptus coolabah* Blakely & Jacobs Blakely & Jacobs |
| 12 | 1 | -37.5069 | 145.9311 | VIC-Givnish | *Eucalyptus regnans* F.Muell. |
| 13 | 2 | -37.5009 | 145.9352 | VIC-Givnish | *Eucalyptus regnans* F.Muell. *Eucalyptus obliqua* L'Hér. |
| 14 | 5 | -36.9351 | 145.6596 | VIC-Givnish | *Eucalyptus dives* Schauer |
| 15 | 6 | -37.278 | 145.8764 | VIC-Givnish | *Eucalyptus macrorhyncha* F.Muell. |
| 16 | 7 | -37.3085 | 145.8909 | VIC-Givnish | *Eucalyptus viminalis* Labill. |
| 17 | 8 | -37.3203 | 145.8966 | VIC-Givnish | *Eucalyptus viminalis* Labill. |
| 18 | 9 | -37.3677 | 145.9211 | VIC-Givnish | *Eucalyptus regnans* F.Muell. |
| 19 | 10 | -37.4237 | 145.9412 | VIC-Givnish | *Eucalyptus obliqua* L'Hér. |
| 20 | 13 | -36.7952 | 144.5445 | VIC-Givnish | *Eucalyptus goniocalyx* F.Muell. ex Miq. |
| 21 | 15 | -36.5647 | 144.373 | VIC-Givnish | *Eucalyptus sideroxylon* A.Cunn. ex Woolls |
| 22 | 16 | -36.6055 | 143.7965 | VIC-Givnish | *Eucalyptus sideroxylon* A.Cunn. ex Woolls |
| 23 | 17 | -36.6396 | 143.2644 | VIC-Givnish | *Eucalyptus macrorhyncha* F.Muell. |
| 24 | 18 | -36.4657 | 141.6565 | VIC-Givnish | *Eucalyptus arenacea* Marginson & Ladiges |
| 25 | 19 | -34.7579 | 142.3273 | VIC-Givnish | *Eucalyptus incrassata* Labill. |
| 26 | 20 | -35.2598 | 142.4044 | VIC-Givnish | *Eucalyptus dumosa* A.Cunn. ex J.Oxley |
| 27 | 21 | -35.4432 | 142.4282 | VIC-Givnish | *Eucalyptus dumosa* A.Cunn. ex J.Oxley |
| 28 | O | -37.5542 | 145.8809 | VIC-Givnish | *Eucalyptus regnans* F.Muell. |
| 29 | A | -37.6564 | 145.7378 | VIC-Givnish | *Eucalyptus regnans* F.Muell. |
| 30 | K | -37.426 | 145.1873 | VIC-Givnish | *Eucalyptus regnans* F.Muell. |
| 31 | X | -37.2896 | 145.8791 | VIC-Givnish | *Eucalyptus macrorhyncha* F.Muell. |
| 32 | Currawinya NP | -28.8 | 144.6 | SE-QLD-Stewart | 26 species sampled,  dominant *Acacia aneura* F.Muell. ex Benth. |
| 33 | Idalia NP | -24.5 | 145 | SE-QLD-Stewart | 44 species sampled,  dominant *Acacia harpophylla* F.Muell. ex Benth. |
| 34 | Bell/Jandowae | -26.8 | 151.5 | SE-QLD-Stewart | 28 species sampled,  open savannah woodland |
| 35 | Mt Berriman | -27.7 | 152.4 | SE-QLD-Stewart | 24 species sampled,  dry rainforest/vine thicket |
| 36 | Gambubal SF | -28.2 | 152.5 | SE-QLD-Stewart | 28 species sampled,  subtropical rainforest |
| 37 | Coominya | -27.2 | 152.5 | SE-QLD-Stewart | 12 species sampled,  open forest - *Eucalyptus* spp dominant |
| 38 | Mt Glorious | -27.3 | 152.7 | SE-QLD-Stewart | 32 species sampled,  subtropical rainforest |
| 39 | Pine Mountain | -27.5 | 152.8 | SE-QLD-Stewart | 57 species sampled,  dry rainforest |
| 40 | Mt Coot-tha | -27.5 | 152.9 | SE-QLD-Stewart | 15 species sampled,  open forest - *Eucalyptus* spp dominant |
| 41 | Beerwah SF | -26.9 | 152.9 | SE-QLD-Stewart | 25 species sampled,  wet coastal heathland |
| 42 | Loganholme | -27.8 | 153.1 | SE-QLD-Stewart | 28 species sampled,  swamp - *Melaleuca* sp dominant |
| 43 | Lamington NP | -28.3 | 153.2 | SE-QLD-Stewart | 29 species sampled,  subtropical rainforest |
| 44 | Tyler Pass | -23.6667 | 132.35 | NAT-Schulze | *Eucalyptus terminalis* F.Muell. |
| 45 | Giles | -25.0333 | 128.3 | NAT-Schulze | *Acacia aneura* F.Muell. ex Benth. *Eucalyptus terminalis* F.Muell. |
| 46 | Giles Jct | -25.1333 | 128.55 | NAT-Schulze | *Allocasuarina decaisneana* (Miq.) L.A.S.Johnson *Eucalyptus terminalis* F.Muell. *Grevillea juniperina* R.Br. |
| 47 | Mt Miller | -25.0667 | 129.5667 | NAT-Schulze | *Acacia aneura* F.Muell. ex Benth. *Eucalyptus gummifera* (Sol. ex Gaertn.) Hochr. *Eucalyptus terminalis* F.Muell. |
| 48 | Olgas | -25.3 | 130.6833 | NAT-Schulze | *Allocasuarina decaisneana* (Miq.) L.A.S.Johnson *Acacia aneura* F.Muell. ex Benth. *Eucalyptus terminalis* F.Muell. |
| 49 | Ayers Rock | -25.3333 | 131.0167 | NAT-Schulze | *Acacia aneura* F.Muell. ex Benth. *Acacia olgana* Maconochie |
| 50 | Sandy Blight | -23.2167 | 129.8833 | NAT-Schulze | *Acacia coriacea* DC. *Eucalyptus terminalis* F.Muell. *Hakea divaricata* L.A.S.Johnson |
| 51 | Kintore | -23.3667 | 129.3667 | NAT-Schulze | *Acacia coriacea* DC. *Eucalyptus terminalis* F.Muell. *Hakea divaricata* L.A.S.Johnson |
| 52 | Tennant Creek 2 | -20.35 | 134.2333 | NAT-Schulze | *Acacia aneura* F.Muell. ex Benth. |
| 53 | Tennant Creek 3 | -21.1333 | 134.15 | NAT-Schulze | *Eucalyptus papuana* F.Muell. *Eucalyptus terminalis* F.Muell. |
| 54 | Mt Sanford | -17.3 | 130.75 | NAT-Schulze | *Bauhinia cunninghamii* (Benth.) Benth. *Corymbia bleeseri* (Blakely) K.D.Hill & L.A.S.Johnson *Eucalyptus brevifolia* F.Muell. *Grevillea dimidiata* F.Muell. *Hakea leucoptera* R.Br. *Terminalia arostrata* Ewart & O.B.Davies *Terminalia canescens* (DC.) Radlk. *Ventilago viminalis* Hook. |
| 55 | Tennant Creek 1 | -17.7333 | 133.6333 | NAT-Schulze | *Eucalyptus papuana* F.Muell. *Eucalyptus setosa* Schauer |
| 56 | Kidman Springs | -16.1167 | 130.9167 | NAT-Schulze | *Acacia colei* Maslin & L.A.J.Thomson *Bauhinia cunninghamii* (Benth.) Benth. *Carissa spinarum* L. *Corymbia bleeseri* (Blakely) K.D.Hill & L.A.S.Johnson *Eucalyptus brevifolia* F.Muell. *Eucalyptus pruinosa* Schauer *Eucalyptus tectifica* F.Muell. *Hakea arborescens* R.Br. *Melaleuca* sp |
| 57 | Victoria | -15.5833 | 131.1 | NAT-Schulze | *Adansonia gregorii* F.Muell. *Erythrophleum chlorostachys* (F.Muell.) Baill. *Eucalyptus miniata* A.Cunn. ex Schauer *Eucalyptus tectifica* F.Muell. |
| 58 | Katherine | -14.3 | 132.0833 | NAT-Schulze | *Acacia dimidiata* Benth. *Brachychiton diversifolius* R.Br. *Brachychiton megaphyllus* Guymer *Buchanania obovata* Engl. *Cochlospermum fraseri* (Hook.) F.Muell. *Erythrophleum chlorostachys* (F.Muell.) Baill. *Eucalyptus confertiflora* Maiden & Blakely *Eucalyptus bleeseri* Blakely *Eucalyptus miniata* A.Cunn. ex Schauer *Eucalyptus tetrodonta* F.Muell. *Grevillea pyramidalis* A.Cunn. ex R.Br. *Lysiphyllum cunninghamii* (Benth.) de Wit *Planchonia careya* (F.Muell.) R.Knuth *Petalostigma quadriloculare* (F.Muell.) Benth. |
| 59 | Kapalga | -12.6833 | 132.3833 | NAT-Schulze | *Acacia mimula* Pedley *Buchanania obovata* Engl. *Corymbia clavigera* (A.Cunn. ex Schauer) K.D.Hill & L.A.S.Johnson *Corymbia porrecta* (S.T.Blake) K.D.Hill & L.A.S.Johnson *Erythrophleum chlorostachys* (F.Muell.) Baill. *Eucalyptus miniata* A.Cunn. ex Schauer *Eucalyptus tectifica* F.Muell. *Eucalyptus tetrodonta* F.Muell. *Livistona humilis* R.Br. *Planchonia careya* (F.Muell.) R.Knuth *Xanthostemon paradoxus* F.Muell. |
| 60 | Darwin | -12.4167 | 130.8667 | NAT-Schulze | *Anacardium occidentale* L. *Mangifera indica* L. *Garcinia mangostana* L. |
| 61 | Melville | -11.7667 | 130.8667 | NAT-Schulze | *Cochlospermum fraseri* Planch. *Cycas armstrongii* Miq. *Erythrophleum chlorostachys* (F.Muell.) Baill. *Eucalyptus miniata* A.Cunn. ex Schauer *Corymbia nesophila* (Blakely) K.D.Hill & L.A.S.Johnson *Eucalyptus tetrodonta* F.Muell. *Livistona humilis* R.Br. *Persoonia falcata* R.Br. |
| 62 | Walpole | -34.97 | 116.791 | WA-Schulze | *Eucalyptus jacksonii* Maiden |
| 63 | Denmark | -34.966 | 117.289 | WA-Schulze | *Eucalyptus diversicolor* F. Muell. |
| 64 | Frankland | -34.911 | 116.689 | WA-Schulze | *Eucalyptus guilfoylei* Maiden |
| 65 | Mt Baker | -34.744 | 117.506 | WA-Schulze | *Eucalyptus wandoo* Blakely wandoo |
| 66 | Kalcup Rd | -34.53 | 116.01 | WA-Schulze | *Corymbia calophylla* (Lindl.) K.D. Hill & L.A.S. Johnson *Eucalyptus marginata* Donn ex Smith |
| 67 | Warren | -34.492 | 115.952 | WA-Schulze | *Corymbia calophylla* (Lindl.) K.D. Hill & L.A.S. Johnson *Eucalyptus diversicolor* F. Muell. |
| 68 | Stirling Range | -34.379 | 117.78 | WA-Schulze | *Corymbia calophylla* (Lindl.) K.D. Hill & L.A.S. Johnson *Eucalyptus marginata* Donn ex Smith *Eucalyptus wandoo* Blakely wandoo |
| 69 | Amelup | -34.149 | 118.235 | WA-Schulze | *Eucalyptus longicornis* (F. Muell.) F. Muell. ex Maiden |
| 70 | Bordon | -33.919 | 118.329 | WA-Schulze | *Eucalyptus phaenophylla* Brooker & Hopper |
| 71 | Nanicup | -33.711 | 118.365 | WA-Schulze | *Eucalyptus astringens* (Maiden) Maiden *Eucalyptus phaenophylla* Brooker & Hopper |
| 72 | Pingrup | -33.598 | 118.441 | WA-Schulze | *Eucalyptus longicornis* (F. Muell.) F. Muell. ex Maiden *Eucalyptus salmonophloia* F. Muell. |
| 73 | Ludlow | -33.581 | 115.499 | WA-Schulze | *Eucalyptus gomphocephala* DC. |
| 74 | Chinocup | -33.548 | 118.378 | WA-Schulze | *Eucalyptus gratiae* Brooker *Eucalyptus tenera* L. Johnson & K. Hill |
| 75 | Lake Grace | -33.382 | 118.51 | WA-Schulze | *Eucalyptus flocktoniae* (Maiden) Maiden flocktoniae *Eucalyptus pileata* Blakely *Eucalyptus ravida* L.A.S.Johnson & K.D.Hill |
| 76 | Grace camp | -33.165 | 118.473 | WA-Schulze | *Eucalyptus ravida* L.A.S.Johnson & K.D.Hill *Eucalyptus salmonophloia* F. Muell. *Eucalyptus urna* Nicolle |
| 77 | Myalup | -33.007 | 115.743 | WA-Schulze | *Eucalyptus gomphocephala* DC. |
| 78 | Dragon Rock | -32.73 | 119.005 | WA-Schulze | *Eucalyptus astringens* (Maiden) Maiden *Eucalyptus flocktoniae* (Maiden) Maiden flocktoniae *Eucalyptus phaenophylla* Brooker & Hopper |
| 79 | Dragon North | -32.671 | 118.999 | WA-Schulze | *Eucalyptus latens* Brooker |
| 80 | Jilakin Rock | -32.665 | 118.326 | WA-Schulze | *Eucalyptus gratiae* Brooker *Eucalyptus marginata* Donn ex Smith |
| 81 | Bushfire Rock | -32.426 | 119.356 | WA-Schulze | *Eucalyptus leptopoda* Benth. |
| 82 | Holland 21km | -32.278 | 119.581 | WA-Schulze | *Eucalyptus calycogona* Turcz. *Eucalyptus flocktoniae* (Maiden) Maiden flocktoniae *Eucalyptus livida* Brooker & Hopper *Eucalyptus pileata* Blakely *Eucalyptus ravida* L.A.S.Johnson & K.D.Hill *Eucalyptus salmonophloia* F. Muell. *Eucalyptus steedmanii* C. Gardner *Eucalyptus tenera* L. Johnson & K. Hill |
| 83 | Mt Holland | -32.165 | 119.749 | WA-Schulze | *Eucalyptus calycogona* Turcz. *Eucalyptus concinna* Maiden & Blakely *Eucalyptus flocktoniae* (Maiden) Maiden flocktoniae *Eucalyptus livida* Brooker & Hopper *Eucalyptus longicornis* (F. Muell.) F. Muell. ex Maiden *Eucalyptus polita* Brooker & Hopper *Eucalyptus ravida* L.A.S.Johnson & K.D.Hill *Eucalyptus salmonophloia* F. Muell. *Eucalyptus tenera* L. Johnson & K. Hill *Eucalyptus urna* Nicolle |
| 84 | Twin Rock | -32.11 | 118.793 | WA-Schulze | *Eucalyptus calycogona* Turcz. *Eucalyptus capillosa* Brooker & Hopper *Eucalyptus salmonophloia* F. Muell. *Eucalyptus sheathiana* Maiden *Eucalyptus tephroclada* L. Johnson & K. Hill *Eucalyptus wandoo* Blakely wandoo |
| 85 | Holland 69 | -32.05 | 119.903 | WA-Schulze | *Eucalyptus alipes* (L. Johnson & K. Hill) Nicolle & Brooker *Eucalyptus dendrosheath* Nicolle ms *Eucalyptus exigua* Brooker & Hopper *Eucalyptus salicola* Brooker |
| 86 | York | -31.895 | 116.559 | WA-Schulze | *Corymbia calophylla* (Lindl.) K.D. Hill & L.A.S. Johnson *Eucalyptus marginata* Donn ex Smith *Eucalyptus wandoo* Blakely wandoo |
| 87 | Mundaring | -31.891 | 116.249 | WA-Schulze | *Corymbia calophylla* (Lindl.) K.D. Hill & L.A.S. Johnson *Eucalyptus marginata* Donn ex Smith *Eucalyptus patens* Benth. |
| 88 | Rifle range | -31.884 | 116.262 | WA-Schulze | *Eucalyptus wandoo* Blakely wandoo |
| 89 | Holland 107 | -31.877 | 120.189 | WA-Schulze | *Eucalyptus aequioperta* Brooker & Hopper *Eucalyptus salmonophloia* F. Muell. *Eucalyptus tenuis* Brooker & Hopper *Eucalyptus yilgarnensis* (Maiden) Brooker |
| 90 | Holland 128 | -31.78 | 120.343 | WA-Schulze | *Eucalyptus incerata* Brooker & Hopper *Eucalyptus ravida* L.A.S.Johnson & K.D.Hill *Eucalyptus tenuis* Brooker & Hopper *Eucalyptus transcontinentalis* Maiden |
| 91 | Holland 148 | -31.67 | 120.477 | WA-Schulze | *Eucalyptus leptopoda* Benth. *Eucalyptus rigidula* Maiden |
| 92 | Holland 164 | -31.576 | 120.579 | WA-Schulze | *Eucalyptus aspratilis* L. Johnson & K. Hill *Eucalyptus grossa* F.Muell. ex Benth. *Eucalyptus histophylla* Brooker & Hopper *Eucalyptus rigidula* Maiden |
| 93 | Yanchep | -31.545 | 115.675 | WA-Schulze | *Eucalyptus gomphocephala* DC. |
| 94 | Thursday Rock | -31.511 | 120.816 | WA-Schulze | *Eucalyptus gratiae* Brooker *Eucalyptus ravida* L.A.S.Johnson & K.D.Hill *Eucalyptus salmonophloia* F. Muell. |
| 95 | Queen Victoria Rock | -31.191 | 120.942 | WA-Schulze | *Eucalyptus flocktoniae* (Maiden) Maiden flocktoniae *Eucalyptus tenera* L. Johnson & K. Hill |
| 96 | Kalgoorlie | -30.643 | 121.454 | WA-Schulze | *Eucalyptus lesouefii* Maiden *Eucalyptus salmonophloia* F. Muell. |
| 97 | Menzies | -29.905 | 121.119 | WA-Schulze | *Eucalyptus concinna* Maiden & Blakely *Eucalyptus drummondii* Benth. *Eucalyptus jutsonii* Maiden *Eucalyptus leptopoda* Benth. *Eucalyptus rigidula* Maiden *Eucalyptus transcontinentalis* Maiden |
| 98 | Menzies II | -29.761 | 121.063 | WA-Schulze | *Eucalyptus longissima* Nicolle |
| 99 | Lake Ballard | -29.495 | 121.252 | WA-Schulze | *Eucalyptus youngiana* F. Muell. |
| 100 | Bore Brakeaway | -29.243 | 121.256 | WA-Schulze | *Eucalyptus longissima* Nicolle |
| 101 | Quarzripe | -29.242 | 121.252 | WA-Schulze | *Eucalyptus carnea* R.T.Baker *Eucalyptus carnea* × *Eucalyptus* *salubris* F.Muell. *Eucalyptus ravida* L.A.S.Johnson & K.D.Hill |
| 102 | Mt. Morrains | -28.764 | 121.968 | WA-Schulze | *Eucalyptus lucasii* Blakely *Eucalyptus youngiana* F. Muell. |
| 103 | Admuro flat | -28.565 | 120.482 | WA-Schulze | *Eucalyptus gypsophila* Nicolle *Eucalyptus ravida* L.A.S.Johnson & K.D.Hill |
| 104 | Adam Range | -28.401 | 122.568 | WA-Schulze | *Eucalyptus gongylocarpa* Blakely *Eucalyptus rigidula* Maiden *Eucalyptus youngiana* F. Muell. |
| 105 | Warburton Camp | -28.385 | 122.585 | WA-Schulze | *Eucalyptus lucasii* Blakely *Eucalyptus trivalvis* Blakely *Eucalyptus youngiana* F. Muell. |
| 106 | Cosmo Newberry | -28.227 | 122.732 | WA-Schulze | *Eucalyptus concinna* Maiden & Blakely *Eucalyptus gongylocarpa* Blakely *Eucalyptus leptopoda* Benth. *Eucalyptus rigidula* Maiden *Eucalyptus socialis* F. Muell. ex Miq. *Eucalyptus trivalvis* Blakely |
| 107 | Lake Throssel | -27.918 | 123.616 | WA-Schulze | *Eucalyptus concinna* Maiden & Blakely *Eucalyptus eremicola* Boomsma peeneri (Blakely) Nicolle |
| 108 | Jutson well | -27.899 | 123.378 | WA-Schulze | *Eucalyptus ewartiana* Maiden |
| 109 | Tjukayiria Roadhouse | -27.053 | 125.149 | WA-Schulze | *Eucalyptus drummondii* Benth. |
| 110 | Sideroad I | -27.038 | 125.236 | WA-Schulze | *Eucalyptus leptopoda* Benth. *Eucalyptus lucasii* Blakely *Eucalyptus youngiana* F. Muell. |
| 111 | Parallel Rd | -27.038 | 125.241 | WA-Schulze | *Eucalyptus leptopoda* Benth. *Eucalyptus lucasii* Blakely *Eucalyptus youngiana* F. Muell. |
| 112 | Warburton W | -27.008 | 126.25 | WA-Schulze | *Eucalyptus concinna* Maiden & Blakely *Eucalyptus gamophylla* F. Muell. |
| 113 | On Road | -27.008 | 126.252 | WA-Schulze | *Eucalyptus concinna* Maiden & Blakely *Eucalyptus gamophylla* F. Muell. |
| 114 | Lunchplace | -27 | 126.227 | WA-Schulze | *Eucalyptus gongylocarpa* Blakely *Eucalyptus youngiana* F. Muell. |
| 115 | Sanddunes | -26.999 | 125.534 | WA-Schulze | *Corymbia terminalis* (F.Muell.) K.D.Hill & L.A.S.Johnson *Eucalyptus youngiana* F. Muell. |
| 116 | Sanddune II | -26.996 | 125.559 | WA-Schulze | *Corymbia terminalis* (F.Muell.) K.D.Hill & L.A.S.Johnson *Eucalyptus lucasii* Blakely *Eucalyptus victrix* L. Johnson & K. Hill |
| 117 | Sanddune III | -26.996 | 125.594 | WA-Schulze | *Corymbia terminalis* (F.Muell.) K.D.Hill & L.A.S.Johnson *Eucalyptus lucasii* Blakely *Eucalyptus victrix* L. Johnson & K. Hill |
| 118 | Creek | -26.985 | 125.701 | WA-Schulze | *Eucalyptus intertexta* R. Baker |
| 119 | Breakaway | -26.979 | 126.053 | WA-Schulze | *Eucalyptus trivalvis* Blakely *Eucalyptus youngiana* F. Muell. |
| 120 | ConneySue Hwy | -26.543 | 126.402 | WA-Schulze | *Eucalyptus gamophylla* F. Muell. *Eucalyptus socialis* F. Muell. ex Miq. *Eucalyptus youngiana* F. Muell. |
| 121 |  | -26.532 | 126.404 | WA-Schulze | *Eucalyptus mannensis* Boomsma mannensis |
| 122 | ConneySueCamp | -26.427 | 126.413 | WA-Schulze | *Eucalyptus eremicola* Boomsma peeneri (Blakely) Nicolle *Eucalyptus gongylocarpa* Blakely *Eucalyptus mannensis* Boomsma mannensis *Eucalyptus socialis* F. Muell. ex Miq. |
| 123 | Neili Jucn | -26.368 | 126.285 | WA-Schulze | *Eucalyptus gamophylla* F. Muell. |
| 124 | Rd Warburton | -26.235 | 126.276 | WA-Schulze | *Corymbia chippendalei* (D.J. Carr & S.G.M. Carr) K.D. Hill & L.A.S. Johnson |
| 125 | Warburton | -25.881 | 126.913 | WA-Schulze | *Eucalyptus gamophylla* F. Muell. *Eucalyptus oxymitra* Blakely |
| 126 | Warburton II | -25.827 | 127.011 | WA-Schulze | *Eucalyptus kingsmillii* (Maiden) Maiden & Blakely_x_youngiana |
| 127 | Halfway Giles | -25.419 | 127.554 | WA-Schulze | *Corymbia chippendalei* (D.J. Carr & S.G.M. Carr) K.D. Hill & L.A.S. Johnson *Corymbia terminalis* (F.Muell.) K.D.Hill & L.A.S.Johnson *Eucalyptus gamophylla* F. Muell. *Eucalyptus oxymitra* Blakely |
| 128 | Near Giles | -25.298 | 127.86 | WA-Schulze | *Eucalyptus victrix* L. Johnson & K. Hill |
| 129 | Near Mt Olga | -25.228 | 130.58 | WA-Schulze | *Corymbia terminalis* (F.Muell.) K.D.Hill & L.A.S.Johnson |
| 130 | Giles West | -25.074 | 128.169 | WA-Schulze | *Corumbia aparrerinja* K.D. Hill & L.A.S. Johnson *Corymbia terminalis* (F.Muell.) K.D.Hill & L.A.S.Johnson |
| 131 | Irving River | -25.067 | 129.839 | WA-Schulze | *Eucalyptus mannensis* Boomsma mannensis |
| 132 | Giles | -25.061 | 128.329 | WA-Schulze | *Corumbia aparrerinja* K.D. Hill & L.A.S. Johnson *Eucalyptus intertexta* R. Baker |
| 133 | Docker River E | -25.056 | 129.678 | WA-Schulze | *Eucalyptus gamophylla* F. Muell. *Eucalyptus oxymitra* Blakely |
| 134 | Docker River | -24.871 | 129.057 | WA-Schulze | *Eucalyptus gamophylla* F. Muell. *Eucalyptus gongylocarpa* Blakely |
| 135 | Peterman Range | -24.837 | 128.934 | WA-Schulze | *Corymbia eremaea* (D.J. Carr & S.G.M. Carr) K.D. Hill & L.A.S. Johnson *Eucalyptus oxymitra* Blakely |
| 136 | Howard Springs | -12.4853 | 131.1461 | NAT-Cernusak | *Eucalyptus miniata* A.Cunn. ex Schauer *Eucalyptus tetrodonta* F.Muell. |
| 137 | Adelaide River | -13.0769 | 131.1178 | NAT-Cernusak | *Eucalyptus tectifica* F.Muell. *Corymbia latifolia* (F.Muell.) K.D.Hill & L.A.S.Johnson |
| 138 | Daily river | -14.1592 | 131.3881 | NAT-Cernusak | *Eucalyptus tetrodonta* F.Muell. *Corymbia latifolia* (F.Muell.) K.D.Hill & L.A.S.Johnson |
| 139 | Dry Creek | -15.2589 | 132.3706 | NAT-Cernusak | *Eucalyptus tetrodonta* F.Muell. *Corymbia terminalis* (F.Muell.) K.D.Hill & L.A.S.Johnson |
| 140 | Stuart Plains | -17.1331 | 133.3289 | NAT-Cernusak | *Eucalyptus pruinosa* Schauer *Eucalyptus coolabah* Blakely & Jacobs Blakely & Jacobs |
| 141 | Boulia | -22.9944 | 139.9453 | NAT-Cernusak | *Corymbia terminalis* (F.Muell.) K.D.Hill & L.A.S.Johnson *Corymbia aparrerinja* K.D. Hill & L.A.S. Johnson |
| 142 | 16 degree S | -16.0531 | 133.4263 | NAT-Miller | *Eucalyptus chlorophylla* Brooker & Done *Corymbia confertiflora* (Kippist) K.D.Hill & L.A.S.Johnson *Eucalyptus coolabah* Blakely & Jacobs *Corymbia dichromophloia* (F.Muell.) K.D.Hill & L.A.S.Johnson *Eucalyptus leucophloia* Brooker *Eucalyptus pruinosa* Schauer *Corymbia terminalis* (F.Muell.) K.D.Hill & L.A.S.Johnson |
| 143 | 3 ways | -19.472 | 134.213 | NAT-Miller | *Eucalyptus leucophloia* Brooker *Eucalyptus odontocarpa* F.Muell. *Eucalyptus pachyphylla* F.Muell. *Eucalyptus pruinosa* Schauer *Corymbia terminalis* (F.Muell.) K.D.Hill & L.A.S.Johnson |
| 144 | Acacia shop | -12.827 | 131.134 | NAT-Miller | *Eucalyptus miniata* A.Cunn. ex Schauer *Eucalyptus tetrodonta* F.Muell. |
| 145 | Alice | -24.0415 | 133.6335 | NAT-Miller | *Eucalyptus gamophylla* F. Muell. *Corymbia terminalis* (F.Muell.) K.D.Hill & L.A.S.Johnson |
| 146 | Attack creek | -19.0303 | 134.1492 | NAT-Miller | *Eucalyptus coolabah* Blakely & Jacobs *Corymbia dichromophloia* (F.Muell.) K.D.Hill & L.A.S.Johnson *Eucalyptus leucophloia* Brooker *Eucalyptus odontocarpa* F.Muell. *Eucalyptus pruinosa* Schauer *Corymbia terminalis* (F.Muell.) K.D.Hill & L.A.S.Johnson |
| 147 | Barkley stock route | -17.7735 | 133.653 | NAT-Miller | *Corymbia dichromophloia* (F.Muell.) K.D.Hill & L.A.S.Johnson *Eucalyptus leucophloia* Brooker *Eucalyptus pruinosa* Schauer *Corymbia terminalis* (F.Muell.) K.D.Hill & L.A.S.Johnson |
| 148 | Barrow Ck | -21.5925 | 133.771 | NAT-Miller | *Eucalyptus pachyphylla* F.Muell. *Corymbia terminalis* (F.Muell.) K.D.Hill & L.A.S.Johnson |
| 149 | Buchanan Hwy | -16.5145 | 133.3763 | NAT-Miller | *Corymbia confertiflora* (Kippist) K.D.Hill & L.A.S.Johnson *Eucalyptus coolabah* Blakely & Jacobs *Corymbia dichromophloia* (F.Muell.) K.D.Hill & L.A.S.Johnson *Eucalyptus leucophloia* Brooker *Eucalyptus pruinosa* Schauer *Corymbia terminalis* (F.Muell.) K.D.Hill & L.A.S.Johnson |
| 150 | Central Arnhem Hwy | -14.7457 | 132.839 | NAT-Miller | *Eucalyptus chlorophylla* Brooker & Done *Corymbia confertiflora* (Kippist) K.D.Hill & L.A.S.Johnson *Corymbia dichromophloia* (F.Muell.) K.D.Hill & L.A.S.Johnson *Eucalyptus miniata* A.Cunn. ex Schauer *Eucalyptus tectifica* F.Muell. *Eucalyptus tetrodonta* F.Muell. |
| 151 | Cullen Creek | -14.0148 | 131.9383 | NAT-Miller | *Corymbia confertiflora* (Kippist) K.D.Hill & L.A.S.Johnson *Corymbia dichromophloia* (F.Muell.) K.D.Hill & L.A.S.Johnson *Eucalyptus miniata* A.Cunn. ex Schauer *Eucalyptus tectifica* F.Muell. |
| 152 | Darwin radio beacon | -12.428 | 130.957 | NAT-Miller | *Eucalyptus miniata* A.Cunn. ex Schauer *Eucalyptus tetrodonta* F.Muell. |
| 153 | Devils marbles | -20.6073 | 134.2287 | NAT-Miller | *Eucalyptus odontocarpa* F.Muell. *Eucalyptus pachyphylla* F.Muell. *Corymbia terminalis* (F.Muell.) K.D.Hill & L.A.S.Johnson |
| 154 | Edinburgh Creek | -20.2445 | 134.23 | NAT-Miller | *Eucalyptus leucophloia* Brooker *Eucalyptus odontocarpa* F.Muell. *Eucalyptus pachyphylla* F.Muell. *Corymbia terminalis* (F.Muell.) K.D.Hill & L.A.S.Johnson |
| 155 | Edith Farms | -14.3042 | 132.0964 | NAT-Miller | *Corymbia confertiflora* (Kippist) K.D.Hill & L.A.S.Johnson *Corymbia dichromophloia* (F.Muell.) K.D.Hill & L.A.S.Johnson *Eucalyptus miniata* A.Cunn. ex Schauer *Eucalyptus tectifica* F.Muell. *Eucalyptus tetrodonta* F.Muell. |
| 156 | Elsey River | -15.1688 | 133.0943 | NAT-Miller | *Eucalyptus chlorophylla* Brooker & Done *Corymbia confertiflora* (Kippist) K.D.Hill & L.A.S.Johnson *Corymbia dichromophloia* (F.Muell.) K.D.Hill & L.A.S.Johnson *Eucalyptus miniata* A.Cunn. ex Schauer *Eucalyptus pruinosa* Schauer *Eucalyptus tetrodonta* F.Muell. |
| 157 | Erldunda | -24.915 | 133.198 | NAT-Miller | *Eucalyptus gamophylla* F. Muell. |
| 158 | Hansens Well | -21.938 | 133.526 | NAT-Miller | *Eucalyptus pachyphylla* F.Muell. *Corymbia terminalis* (F.Muell.) K.D.Hill & L.A.S.Johnson |
| 159 | Hayes Creek | -13.6765 | 131.64 | NAT-Miller | *Corymbia confertiflora* (Kippist) K.D.Hill & L.A.S.Johnson *Corymbia dichromophloia* (F.Muell.) K.D.Hill & L.A.S.Johnson *Eucalyptus miniata* A.Cunn. ex Schauer *Eucalyptus tetrodonta* F.Muell. |
| 160 | Jirns Place | -24.345 | 133.448 | NAT-Miller | *Eucalyptus gamophylla* F. Muell. *Corymbia terminalis* (F.Muell.) K.D.Hill & L.A.S.Johnson |
| 161 | Katherine | -14.543 | 132.4536 | NAT-Miller | *Corymbia confertiflora* (Kippist) K.D.Hill & L.A.S.Johnson *Corymbia dichromophloia* (F.Muell.) K.D.Hill & L.A.S.Johnson *Eucalyptus miniata* A.Cunn. ex Schauer *Eucalyptus tectifica* F.Muell. *Eucalyptus tetrodonta* F.Muell. |
| 162 | Larrimah | -15.6276 | 133.3088 | NAT-Miller | *Eucalyptus chlorophylla* Brooker & Done *Corymbia confertiflora* (Kippist) K.D.Hill & L.A.S.Johnson *Eucalyptus coolabah* Blakely & Jacobs *Corymbia dichromophloia* (F.Muell.) K.D.Hill & L.A.S.Johnson *Eucalyptus leucophloia* Brooker *Eucalyptus miniata* A.Cunn. ex Schauer *Eucalyptus pruinosa* Schauer *Corymbia terminalis* (F.Muell.) K.D.Hill & L.A.S.Johnson *Eucalyptus tetrodonta* F.Muell. |
| 163 | Muckaty Stn | -18.632 | 133.947 | NAT-Miller | *Eucalyptus coolabah* Blakely & Jacobs *Corymbia dichromophloia* (F.Muell.) K.D.Hill & L.A.S.Johnson *Eucalyptus leucophloia* Brooker *Eucalyptus odontocarpa* F.Muell. *Eucalyptus pruinosa* Schauer *Corymbia terminalis* (F.Muell.) K.D.Hill & L.A.S.Johnson |
| 164 | Native Gap | -22.825 | 133.4355 | NAT-Miller | *Eucalyptus gamophylla* F. Muell. *Corymbia terminalis* (F.Muell.) K.D.Hill & L.A.S.Johnson |
| 165 | Newcastle Water | -17.3658 | 133.4625 | NAT-Miller | *Eucalyptus coolabah* Blakely & Jacobs *Corymbia dichromophloia* (F.Muell.) K.D.Hill & L.A.S.Johnson *Eucalyptus pruinosa* Schauer *Corymbia terminalis* (F.Muell.) K.D.Hill & L.A.S.Johnson |
| 166 | Plenty Hwy | -23.131 | 133.697 | NAT-Miller | *Corymbia terminalis* (F.Muell.) K.D.Hill & L.A.S.Johnson |
| 167 | Renner Springs | -18.2634 | 133.7706 | NAT-Miller | *Eucalyptus coolabah* Blakely & Jacobs *Corymbia dichromophloia* (F.Muell.) K.D.Hill & L.A.S.Johnson *Eucalyptus leucophloia* Brooker *Eucalyptus pruinosa* Schauer *Corymbia terminalis* (F.Muell.) K.D.Hill & L.A.S.Johnson |
| 168 | Shenandoah | -16.8773 | 133.4253 | NAT-Miller | *Corymbia confertiflora* (Kippist) K.D.Hill & L.A.S.Johnson *Eucalyptus coolabah* Blakely & Jacobs *Corymbia dichromophloia* (F.Muell.) K.D.Hill & L.A.S.Johnson |
| 169 | Site 3 | -13.072 | 131.1025 | NAT-Miller | *Eucalyptus miniata* A.Cunn. ex Schauer *Eucalyptus tectifica* F.Muell. |
| 170 | Site 4 | -13.4547 | 131.3223 | NAT-Miller | *Eucalyptus miniata* A.Cunn. ex Schauer *Eucalyptus tectifica* F.Muell. *Eucalyptus tetrodonta* F.Muell. |
| 171 | Tanami Rd | -23.471 | 133.843 | NAT-Miller | *Corymbia terminalis* (F.Muell.) K.D.Hill & L.A.S.Johnson |
| 172 | Tea Tree | -22.305 | 133.41 | NAT-Miller | *Corymbia terminalis* (F.Muell.) K.D.Hill & L.A.S.Johnson |
| 173 | Tennant Creek | -19.7378 | 134.1835 | NAT-Miller | *Eucalyptus leucophloia* Brooker *Eucalyptus odontocarpa* F.Muell. *Eucalyptus pruinosa* Schauer *Corymbia terminalis* (F.Muell.) K.D.Hill & L.A.S.Johnson |
| 174 | Warribri | -20.8427 | 134.1237 | NAT-Miller | *Eucalyptus gamophylla* F. Muell. *Eucalyptus pachyphylla* F.Muell. *Corymbia terminalis* (F.Muell.) K.D.Hill & L.A.S.Johnson |
